# Yiqi Huoxue Yangyin Decoction attenuates diabetic nephropathy in *db/db* mice by modulating METTL3-mediated m6A methylation of mTOR to restore podocyte autophagy

**DOI:** 10.3389/fphar.2026.1783423

**Published:** 2026-04-07

**Authors:** JinXing He, WenDi Niu, JiaHui Qian, JianHong Jin, Fei Pan, XueQian Peng, Ai Mi, WenHong Liu, ZhiWei Xu, YanFang Yang, Hui Wang

**Affiliations:** 1 School of Pharmaceutical Sciences, Zhejiang Chinese Medical University, Hangzhou, Zhejiang, China; 2 The Hangzhou Traditional Chinese Medicine Hospital, Hangzhou, Zhejiang, China; 3 Hangzhou Lin’an Hospital of Traditional Chinese Medicine, Hangzhou, Zhejiang, China; 4 Jinhua Academy, Zhejiang Chinese Medical University, Jinhua, Zhejiang, China; 5 School of Basic Medical Sciences, Zhejiang Chinese Medical University, Hangzhou, Zhejiang, China

**Keywords:** autophagy, diabetic nephropathy, MPC-5 cell, N6-methyladenosine, Yiqi Huoxue Yangyin Decoction

## Abstract

**Background:**

Diabetic nephropathy (DN) is a major complication of diabetes with limited therapeutic options. Yiqi Huoxue Yangyin Decoction (YHY), a traditional Chinese medicine formula designed to treat the TCM syndrome of “Qi and Yin deficiency with blood stasis” often observed in DN, has shown clinical potential. However, its precise mechanism of action, particularly concerning the regulation of podocyte autophagy, remains unclear.

**Methods:**

The chemical profile of YHY was characterized using ultra-performance liquid chromatography-tandem mass spectrometry (UPLC-MS/MS). The therapeutic effects and mechanisms were evaluated in a *db/db* mouse model of DN and in high glucose-stimulated MPC-5 podocytes. Key assessments included renal function, podocyte injury markers, autophagy flux, and the activity of the METTL3-m6A-mTOR pathway.

**Results:**

UPLC-MS/MS analysis successfully identified multiple chemical metabolites in YHY, providing a comprehensive phytochemical profile of the formula. Treatment with YHY significantly ameliorated renal dysfunction and attenuated podocyte injury in *db/db* mice. Both *in vivo* and *in vitro* models exhibited impaired autophagy and hyperactivated mTOR signaling under diabetic conditions. Crucially, YHY treatment inhibited METTL3 expression and m6A methylation levels, leading to decreased mTOR mRNA stability and protein expression, which subsequently restored autophagic activity.

**Conclusion:**

YHY, a traditional Chinese medicine formula with defined chemical metabolites, ameliorates podocyte damage in DN by activating autophagy via the METTL3-m6A-mTOR signaling pathway. This study provides novel insights into the epigenetic regulation of DN and underscores the potential of YHY as a therapeutic agent for this condition.

## Graphical Abstract

**Figure:**
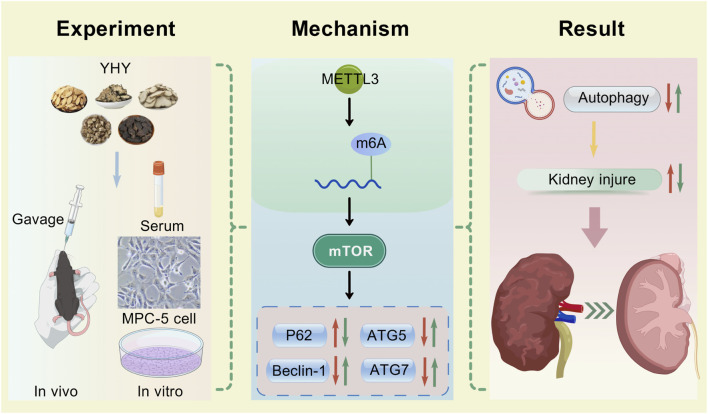

## 1 Introduction

Diabetic nephropathy (DN), a severe complication of diabetes (Martínez-Castelao et al., 2023), has emerged as the primary cause of chronic kidney disease and end-stage renal disease globally, representing over 40% of end-stage renal disease cases worldwide and posing a significant mortality risk (Ma et al., 2025). Epidemiological studies suggest that 30%–40% of the approximately 537 million adults with diabetes may develop DN (Tuttle et al., 2022). As the prevalence of DN rises, novel therapeutic options are consistently being developed. The current management of DN employs a multifaceted pharmacological strategy that integrates glycaemic control (e.g., sodium-glucose cotransporter 2 [SGLT2] inhibitors, glucagon-like peptide-1 [GLP-1] receptor agonists), renin-angiotensin-aldosterone system inhibition (e.g., Angiotensin Converting Enzyme [ACE] inhibitors, angiotensin receptor blocker [ARBs]), and metabolic modulation (e.g., statins). Although these therapies may decelerate disease progression, their nephroprotective benefits are constrained, resulting in many patients enduring a continuous deterioration of renal function. This therapeutic gap highlights the pressing necessity to clarify new molecular pathways underlying DN pathogenesis and to determine specific treatment methods (Chaudhuri et al., 2022).

Podocyte injury plays a critical initiating role in DN pathogenesis (Zhong et al., 2024). Podocytes, as terminally developed glomerular epithelial cells, sustain the integrity of the glomerular filtration barrier by their distinctive cytoskeletal architecture (Cunanan et al., 2025). In a high-glucose microenvironment, podocytes exhibit heightened sensitivity to metabolic dysfunctions, resulting in cytoskeletal disintegration and downregulation of essential podocyte membrane proteins (e.g., Nephrin, Podocin), expedited apoptosis, and irreversible detachment due to dedifferentiation, impaired autophagy, and the accumulation of senescent phenotypes (Li L. et al., 2025; Li et al., 2023). These alterations occur prior to conventional pathological indicators (such as glomerular basement membrane thickening and mesangial enlargement) and directly result in the rupture of the filtration barrier, clinically evident as the development from microalbuminuria to overt proteinuria (Zhang et al., 2024). The loss of podocytes is becoming acknowledged as a pivotal factor in glomerulosclerosis, with its irreparable character propelling permanent damage progression, highlighting its significance in targeted therapy for DN. Consequently, it is imperative to investigate the function and mechanism of podocytes in the initiation of DN.

Autophagy is a conserved degradation mechanism that protects podocytes by clearing damaged proteins and organelles under diabetic conditions. Its dysregulation contributes to DN progression, characterized by podocyte injury, lysosomal dysfunction (Wu et al., 2021), and cellular senescence (Zhao et al., 2024). The mTOR signaling pathway, a key negative regulator of autophagy (Shi et al., 2025), is hyperactivated in DN, suppressing autophagic flux and exacerbating cellular damage (Liu et al., 2024). Enhancing autophagy has been shown to mitigate podocyte injury and proteinuria (Li et al., 2024). Emerging evidence links autophagy to N6-methyladenosine (m6A) RNA modification, a dynamic epigenetic mechanism regulating gene expression (Zhang and Wang, 2023). The m6A modification, controlled by methyltransferases (Methyltransferase like 3 [METTL3] and Methyltransferase like 14 [METTL14], demethylases, and binding proteins (Lin et al., 2023), can modulate autophagy-related genes in podocytes and delay DN progression (Zhang M. et al., 2025). Notably, METTL3 has been implicated in regulating mTOR signaling (Cheng et al., 2022), suggesting a potential METTL3-m6A-mTOR axis connecting epitranscriptomics to autophagic dysfunction in DN.

Traditional Chinese Medicine (TCM) formulas, characterized by their multi-ingredient and multi-target profiles, have long served as a valuable source for drug discovery. In TCM, Yuye Decoction and Siwu Decoction have been widely used in patients with DN. Yuye Decoction, derived from Zhang Xichun’s Qing Dynasty work “Medical Records of Integrating Chinese and Western Medicine”, represents a fundamental prescription for early-stage DN, with documented glucose-lowering efficacy in both clinical and animal experimental settings (He et al., 2023). Siwu Decoction, from “Taiping Huimen Heji Jufang”, providing valuable adjunctive therapy in advanced DN where blood stasis syndrome and renal anemia predominate. Based on modified applications of these classical formulations, our research group developed the Yiqi Huoxue Yangyin Decoction (YHY). YHY comprises five botanical drugs: Sheng Huangqi (*Astragalus membranaceus (Fisch.) Bge. var. mongholicus (Bge.) Hsiao* root), Chuanxiong (*Ligusticum chuanxiong Hort.* rhizome), Gegen (*Pueraria lobata (Willd.) Ohwi* root), Shengdi (*Rehmannia glutinosa Libosch.* root), and Tianhuafen (*Trichosanthes kirilowii Maxim.* root). This formulation is designed to replenish qi and yin, activate blood circulation, and clear heat, thereby targeting the core pathogenesis of DN, which is characterized in TCM as root deficiency (qi and yin depletion) with branch excess (blood stasis and internal heat). Within this formulation, Astragalus membranaceus acts as the sovereign herb to tonify spleen qi, supported by Rehmannia glutinosa as the deputy to nourish yin and clear heat, while Ligusticum chuanxiong, Pueraria lobata, and Trichosanthes kirilowii serve as assistants to promote blood circulation, enhance fluid production, and resolve residual heat. YHY’s distinctive polypharmacological profile exemplifies the capacity of botanical medicine to achieve individualized therapeutic outcomes through multi-target modulation, yet despite previous demonstrations of its hypoglycemic and renoprotective effects in DN models, the specific autophagy-mediated mechanisms underlying its effects remain incompletely characterized (Deng et al., 2018).

Hence, this study first conducted an extensive network pharmacology analysis to uncover its potential targets, which was further integrated with RNA-seq data for mechanistic exploration. The chemical profile of YHY was characterized using UPLC-MS/MS. Then, through both *in vivo* experiments employing *db/db* mice and *in vitro* studies using high glucose-induced MPC-5 podocytes, we deciphered the renoprotective mechanism of YHY, focusing on the novel METTL3-m6A-mTOR-autophagy pathway.

## 2 Methods

### 2.1 Experimental drugs

The botanical formulation YHY comprises five constituent medicinal plants: Sheng Huangqi (*Astragalus membranaceus (Fisch.) Bge. var. mongholicus (Bge.) Hsiao* root), Chuanxiong (*Ligusticum chuanxiong Hort.* rhizome), Gegen (*Pueraria lobata (Willd.) Ohwi* root), Shengdi (*Rehmannia glutinosa Libosch.* root), Tianhuafen (*Trichosanthes kirilowii Maxim.* root). All botanical materials were procured from Zhejiang Chinese Medical University Herbal Pieces Co., Ltd. (Hangzhou, China).

YHY extract preparation. The YHY formulation (24.0 g Huangqi, 6.0 g Chuanxiong, 12.0 g Shengdi, 12.0 g Gegen, 6.0 g Tianhuafen) was macerated in 1.0 L ultrapure water (25 °C, 30 min), then decocted with an additional 3.0 L ultrapure water. After filtration (0.22 μm), the marc was re-extracted under identical conditions. The combined filtrates were concentrated via rotary evaporation (60 °C, 80 rpm) to 3 g crude drug/mL, aliquoted, and either used immediately or lyophilized and stored at −80 °C. For comparative purposes, dapagliflozin (AstraZeneca, UK) was prepared as an aqueous solution (1.3 mg/mL).

YHY-containing serum preparation. Healthy SD rats received daily high-dose YHY extract by gavage for 4 days; controls received an equal volume of normal saline. At 1–2 h after the final gavage, blood was collected from the abdominal aorta, allowed to clot, and centrifuged (3,000 rpm, 10–15 min) to separate serum. Sera were inactivated (56 °C, 30 min), filter-sterilized (0.22 μm), aliquoted, and stored at −80 °C.

### 2.2 Identification of detected metabolites in YHY and its drug-containing serum

Lyophilized YHY extract was reconstituted in 50% methanol-water (10 mg/mL), centrifuged (12,000 × g, 5 min), and filtered (0.22 μm PES membrane). Serum samples (0.1 mL) were precipitated with four volumes of methanol, vortexed (2 min), and centrifuged (12,000 rpm, 15 min). Chromatographic separation was performed on a CORTECS T3 C18 column (2.1 mm × 150 mm, 1.6 μm; 35 °C) using 0.1% aqueous formic acid (A) and acetonitrile (B) at 0.3 mL/min with gradient elution: 0–2 min, 5% B; 2–32 min, 5%–100% B; 32–33 min, 100% B; 33.5–35 min, 5% B. Injection volume was 2 μL. Mass spectrometric detection employed ESI in dual-polarity MSE mode (m/z 50–1,200; scan time 0.2 s) with collision energy ramping (low 6 V; high 15–45 V). Ionization parameters: capillary ±3.0 kV; cone voltage 40 V; source offset 80 V; desolvation temperature 500 °C (positive)/400 °C (negative); desolvation gas flow 1000 L/h (positive)/800 L/h (negative); nebulizer gas 6.5 bar. Leucine enkephalin was used for real-time mass correction. LC-MS data were processed using Progenesis Qi; compounds were identified by database matching (*P* < 0.05, mass deviation < ±5 ppm).

### 2.3 Mice and treatments

Male leptin receptor-deficient *db/db* mice (*BKS.Cg-Lepr<db>/+*), with heterozygous littermates (*db/m*) as non-diabetic controls, were used to model type 2 diabetes and DN. All 8-week-old mice were obtained from GemPharmatech Co., Ltd. (Nanjing, China) and housed under specific pathogen-free conditions (20 °C–25 °C, 40%–70% humidity, 12-h light/dark cycle) at the Zhejiang Chinese Medical University Experimental Animal Research Centre. After 1 week of acclimatization, *db/db* mice were stratified by blood glucose and body weight, and randomly assigned to five groups (n = 10 per group): untreated diabetic model (Model), low-dose YHY (YHY-L, 0.4875 g extract/kg/day), medium-dose YHY (YHY-M, 0.975 g extract/kg/day), high-dose YHY (YHY-H, 1.95 g extract/kg/day), and dapagliflozin positive control (1.3 mg/kg/day). Non-diabetic *db/m* mice served as healthy controls (Ctrl). The lowest dose corresponds to the human clinical equivalent dose calculated from 60 g crude drug/day for a 60 kg adult using body surface area normalization (Reagan-Shaw et al., 2008); the medium and high doses represent 2- and 4-fold multiples to establish a dose-response relationship. Treatments were administered once daily by oral gavage (50 µL/10 g body weight) for 4 weeks: YHY groups received the extract at designated doses, Model and Ctrl groups received vehicle (purified water), and the positive control group received dapagliflozin. Body weight and fasting blood glucose (6-h fast) were measured weekly. At week 4, 24-h urine samples were collected using metabolic cages (Tecniplast, Italy) for proteinuria and albuminuria assessment. Mice were then euthanized under deep CO_2_ anesthesia (2–5 L/min) per AVMA Guidelines (2020), and tissues were harvested for further analysis.

All procedures were performed in compliance with the *Regulations on the Administration of Laboratory Animals* and received approval from the Zhejiang Chinese Medical University Institutional Animal Care and Use Committee (Protocol No. IACUC-20240513–32).

### 2.4 Transcriptomics analysis

Kidney tissue samples from four mice per group (control, model, and YHY) were collected for total RNA extraction. Following mRNA enrichment and fragmentation, reverse transcription was performed to synthesize cDNA. Fragments sized 370–420 bp were selected via AMPure XP beads, PCR-amplified, and purified to construct the sequencing library. Libraries passing quality control were pooled and sequenced on the Illumina platform. After quality filtering of raw reads, differential expression analysis was conducted using R packages. Differentially expressed genes (DEGs) were defined by a fold change >1.2 and a *P*-value <0.05. The raw data have been deposited in the GEO database under accession number GSE315612.

### 2.5 GEO database mining

GSE96804 and GSE30529 were selected as core datasets from the GEO database for DN. Using R 4.4.3, differentially expressed targets between DN and normal samples were screened (|log^2^FC| ≥ 0.8, *P* < 0.05), and their intersection was imported into the STRING database (*Homo sapiens*). The HomoSapines mode is selected for analysis. By hiding the unconnected nodes in the network graph, the core targets are further screened.

### 2.6 Serum insulin and glycated hemoglobin quantification

Blood samples were collected and processed at the Animal Experiment Center of Zhejiang Chinese Medical University. Glycated hemoglobin (HbA1c) levels were determined using a high-throughput four-channel automated analyzer (BioHermes WQ-4000, Wuxi BioHermes Bio & Medical Technology Co., Ltd., China). Serum insulin concentrations were quantified using a commercially available mouse-specific enzyme-linked immunosorbent assay (ELISA) kit (Mouse Insulin ELISA Kit, Nanjing Jiancheng Bioengineering Institute, China) following the manufacturer’s protocol.

### 2.7 Histopathological evaluation

Following the experimental protocol, bilateral kidneys were collected. For histology, tissues were fixed in 4% paraformaldehyde (0.1 M phosphate buffer, pH 7.4; Lanjieke Technology Co., Ltd., Beijing, China) for 24 h. After routine processing, 4-μm serial sections were stained with Hematoxylin and Eosin (HE), Periodic Acid-Schiff (PAS), and Masson’s trichrome. Renal pathology was assessed using a semi-quantitative scoring system (0–4) based on the percentage of affected area: 0 = normal; 1 = < 25%; 2 = 25–50%; 3 = 50–75%; 4 = > 75%. Glomerular injury scores were averaged from multiple measurements. For fibrosis quantification, Masson’s trichrome-stained sections were analyzed using ImageJ. Collagen-positive areas (aniline blue signal) were isolated via color deconvolution, thresholded, and expressed as a percentage of total tissue area.

### 2.8 Ultrastructural analysis by transmission electron microscopy

For ultrastructural evaluation, renal cortical tissue (1 mm^3^) was fixed in 2.5% glutaraldehyde (in 0.1 M phosphate buffer, pH 7.4; Sinopharm, Beijing, China) at 4 °C for 24 h, post-fixed, and embedded in epoxy resin (Epon 812). Ultrathin sections (70 nm) were mounted on copper grids, stained with 1% uranyl acetate (30 min) and 0.5% lead citrate (10 min), and examined under a transmission electron microscope.

### 2.9 Immunohistochemical and immunofluorescence analysis

Paraffin-embedded kidney sections were processed for antigen retrieval after deparaffinization and rehydration. For immunohistochemistry, sections were blocked with 10% normal goat serum and incubated overnight at 4 °C with primary antibodies against P62/SQSTM1 (1:1,000; ab109012, Abcam), ULK1 (1:100; A8529, ABclonal), and phospho-mTOR (1:500; ab109268, Abcam). Following HRP-conjugated secondary antibody incubation, signals were developed with DAB and counterstained with hematoxylin. For immunofluorescence, LC3B antibody (1:1,000; ab192890, Abcam) was used, followed by Alexa Fluor 488-conjugated secondary antibody and DAPI nuclear staining. All images were captured via confocal microscopy and analyzed with ImageJ using color deconvolution for IHC and puncta quantification for IF.

### 2.10 Cell culture

Mouse podocyte cells (MPC-5) were obtained from iCell Bioscience (Shanghai, China) and cultured in high-glucose DMEM (Gibco, NY, USA). To evaluate the therapeutic effects of YHY, MPC-5 cells were treated with high glucose (HG; 55 mM, 48 h) in the presence of 10% blank or YHY-containing serum. Based on preliminary dose-response tests, three groups were established: normal glucose (NG; 25 mM glucose +10% blank serum), HG control (55 mM glucose +10% blank serum), and YHY treatment (55 mM glucose +10% YHY-enriched serum). All analyses were performed after treatment completion.

### 2.11 Cell viability assay

Cell proliferation was assessed using the Cell Counting Kit-8 (CCK-8; Selleck Chemicals, TX, USA). MPC-5 cells were seeded in 96-well plates (1 × 10^4^ cells/mL), treated as indicated, and then incubated with 10 µL of CCK-8 reagent in 90 µL of serum-free medium for 2 h at 37 °C. Absorbance was measured at 450 nm using a microplate reader (Thermo Fisher Scientific, MA, USA).

### 2.12 Cell migration analysis

Cell migration was assessed using a wound healing assay. MPC-5 cells were seeded into 6-well plates (2.5 × 10^4^ cells/mL; 2 mL/well) and cultured to confluence. Monolayers were scratched with a sterile 10 μL pipette tip, washed with PBS, and maintained in low-serum (1% FBS) DMEM. Wound closure was imaged by phase-contrast microscopy at 0, 12, and 24 h. The residual wound area was quantified using ImageJ, and migration percentage was calculated relative to the initial area.

### 2.13 Quantitative real-time PCR analysis

Total RNA was extracted from treated MPC-5 cells using TRIzol (Invitrogen). First-strand cDNA was synthesized from 1 μg RNA using a PrimeScript RT kit (TaKaRa; 37 °C for 15 min, 85 °C for 5 s). Quantitative PCR was performed in 20 μL reactions with SYBR Green Master Mix (Selleck Chemicals) on an Eppendorf Mastercycler ep realplex system. Gene-specific primers spanning exon-exon junctions (Supplementary Table 1) were used, with β-actin as the endogenous control. Relative expression was quantified via the 2^^(-△△Ct)^ method and normalized to controls. All reactions were run in technical triplicates.

### 2.14 Western blotting

Protein expression in MPC-5 cells and renal tissues was assessed by Western blotting (n = 3 per group). Equal protein amounts were separated by SDS-PAGE, transferred to nitrocellulose membranes, and blocked with 5% non-fat milk (2 h, RT). After overnight incubation at 4 °C with primary antibodies (Supplementary Table 2), membranes were probed with HRP-conjugated secondary antibodies (1:2000; 2 h, RT). Bands were visualized via enhanced chemiluminescence and quantified densitometrically with ImageJ, normalizing to loading controls.

### 2.15 Quantification of m6A RNA methylation levels

Global m6A methylation levels were quantified using the EpiQuik™ m6A RNA Methylation Quantification Kit (Epigentek). All steps were carried out strictly in accordance with the instructions of the kit.

### 2.16 Cell transfection

Cells in logarithmic growth phase were seeded into 6-well plates (2 × 10^5^ cells/well) in antibiotic-free medium and cultured for 24 h to reach 60%–80% confluence. For transfection, Lipofectamine 3,000 (5 μL; Invitrogen) and plasmid DNA (2.5 μg) or siRNA (50 nM) were separately diluted in 500 μL OPTI-MEM, mixed, incubated for 20 min at RT, and added dropwise to cells. After 6 h, the medium was replaced with fresh antibiotic-free complete medium to minimize cytotoxicity.

### 2.17 Monodansylcadaverine (MDC) staining

Autophagy was detected using an MDC (monodansylcadaverine) staining kit (Beyotime). Cells were incubated with MDC staining solution following the manufacturer’s protocol and then visualized under a fluorescence microscope.

### 2.18 Lysosomal staining using lyso-tracker red fluorescent probe

Cells in logarithmic growth phase were seeded into 24-well plates (5 × 10^4^ cells/well) and cultured for 12 h. After treatment, cells were washed with PBS, stained with 50 nM LysoTracker Red (30 min, 37 °C, dark) and Hoechst 33342 (5 μg/mL, 5 min, RT), and imaged under an inverted fluorescence microscope. Lysosomal fluorescence intensity was quantified with ImageJ and normalized to controls.

### 2.19 Statistical analysis

Data are presented as mean ± SEM. Statistical analyses were performed using GraphPad Prism 9.0. Normality was assessed by the Shapiro-Wilk test. Comparisons between two groups were analyzed by unpaired two-tailed Student’s t-test. For multiple group comparisons, one-way ANOVA followed by Tukey’s *post hoc* test was used, while two-way ANOVA with Bonferroni’s *post hoc* test was applied for analyses involving two independent variables. The threshold for statistical significance was established at three levels: *P* < 0.05 indicating significance, *P* < 0.01 denoting high significance, and *P* < 0.001 representing extreme significance.

## 3 Results

### 3.1 Analysis of the detected metabolites of YHY and its drug-containing serum

**FIGURE 1:**
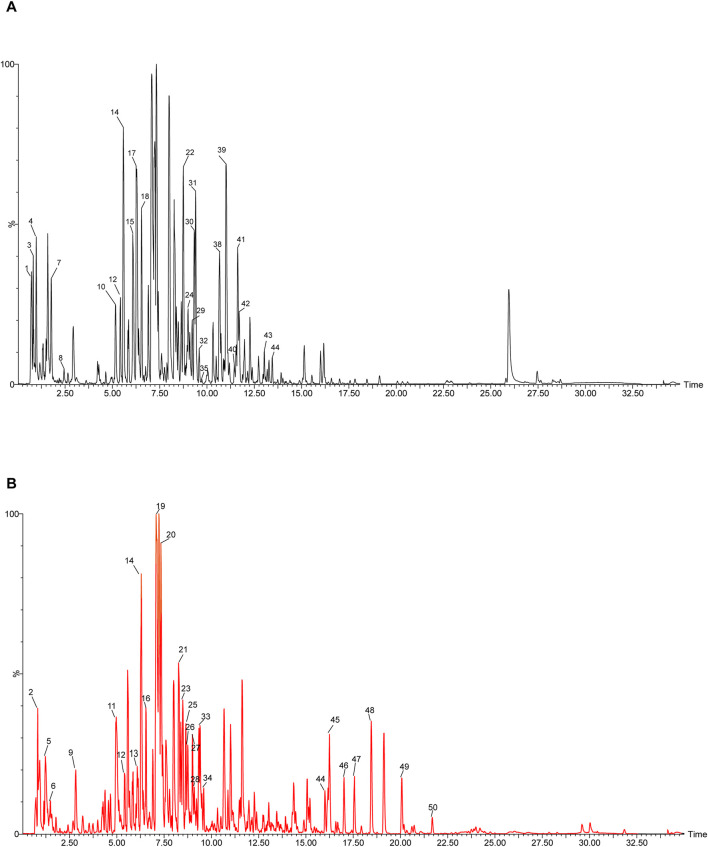
YHY total ion flow diagram. **(A)** The black curve denotes the total ion current for positive ions. **(B)** The red curve denotes the total ion current for negative ions.

The chemical metabolites of YHY were meticulously examined utilising UPLC-MS/MS. It presents representative total ion chromatograms from both positive and negative ionisation modes (Figure 1). Raw mass spectrometric data were processed using UNIFI 2.0 software (Waters Corporation). The specific metabolites are detailed in Supplementary Table 3.

The High-resolution mass spectrometry analysis of drug-containing serum identified 30 metabolites with significantly elevated levels compared to normal serum (Supplementary Figure 1). The major metabolites among these are listed in Supplementary Table 4.

### 3.2 YHY improves blood and urinary glucose levels and renal manifestations of diabetes in *db/db* mice

**FIGURE 2:**
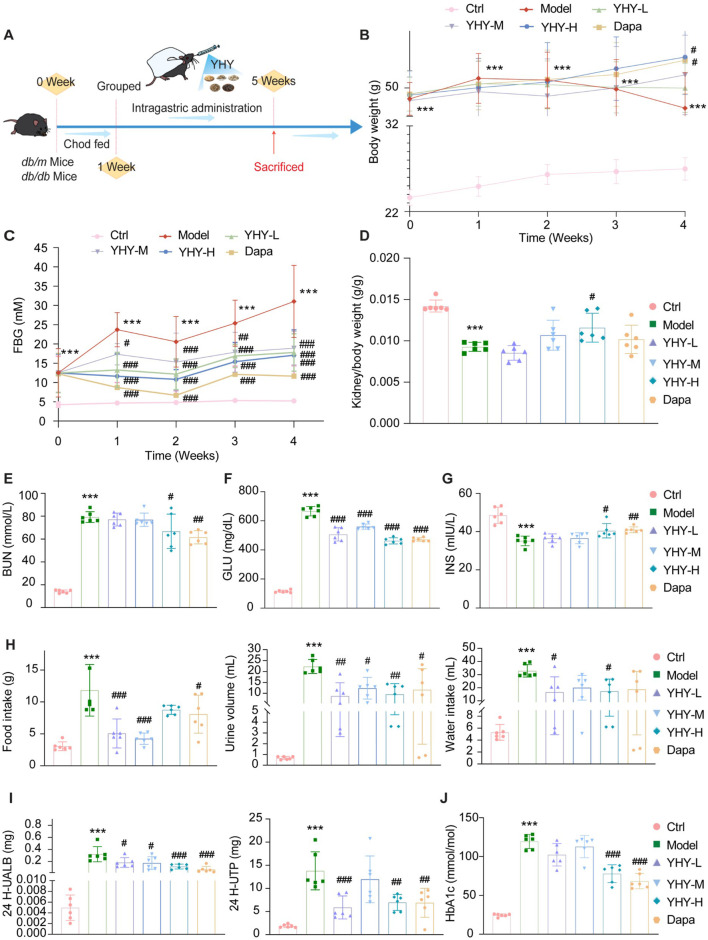
Physical and biochemical analysis of mice. **(A)** Schematic diagram of the animal experiment process. **(B)** Body weight (BW). **(C)** Fasting blood glucose (FBG). **(D)** Kidney/Body weight. **(E)** Blood urea nitrogen (BUN). **(F)** Glucose (GLU). **(G)** Insulin level. **(H)** Food intake, Urine volume, Water intake. **(I)** 24 H-urinary albumin, 24 H-urinary protein quantity. **(J)** Glycated hemoglobin (HbA1c). Data were shown as mean ± SD, ^*^ *P* < 0.05 and ^**^ *P* < 0.01, ^***^ *P* < 0.001 vs. Ctrl; ^#^ *P* < 0.05, ^##^ *P* < 0.01, ^###^ *P* < 0.001 vs. Model.

A comprehensive assessment of the hypoglycemic impact of YHY was performed in accordance with the experimental design illustrated in the schematic diagram (Figure 2A). Multimodal study of blood glucose regulation, pancreatic function, and renal protection revealed a significant enhancement in the metabolism of *db/db* mice: *db/db* mice exhibited significantly elevated fasting blood glucose (FBG) and body weight (BW) relative to the Ctrl group (Figures 2B,C). YHY-H markedly increased the kidney/body weight ratio (Figure 2D), but the YHY-L and YHY-M demonstrated a minimal impact on albumin excretion (Figure 2E). The YHY therapy reduced hyperglycemia (Figure 2F), with YHY-H exhibiting effects similar to Dapa, and normalized plasma insulin levels to values akin to those of Dapa (Figure 2G). YHY ameliorated specific DM irregularities, including polyuria, polydipsia, and polyphagia (Figure 2H), while urinalysis demonstrated reductions in proteinuria and albuminuria (Figure 2I). Both YHY-H and Dapa significantly lowered glycosylated hemoglobin, type A1C (HbA1c) levels (Figure 2J), highlighting YHY’s multifarious benefits on glucose homeostasis, weight regulation, insulin secretion, and renal protection in diabetes.

### 3.3 YHY improves renal dysfunction in *db/db* mice

**FIGURE 3:**
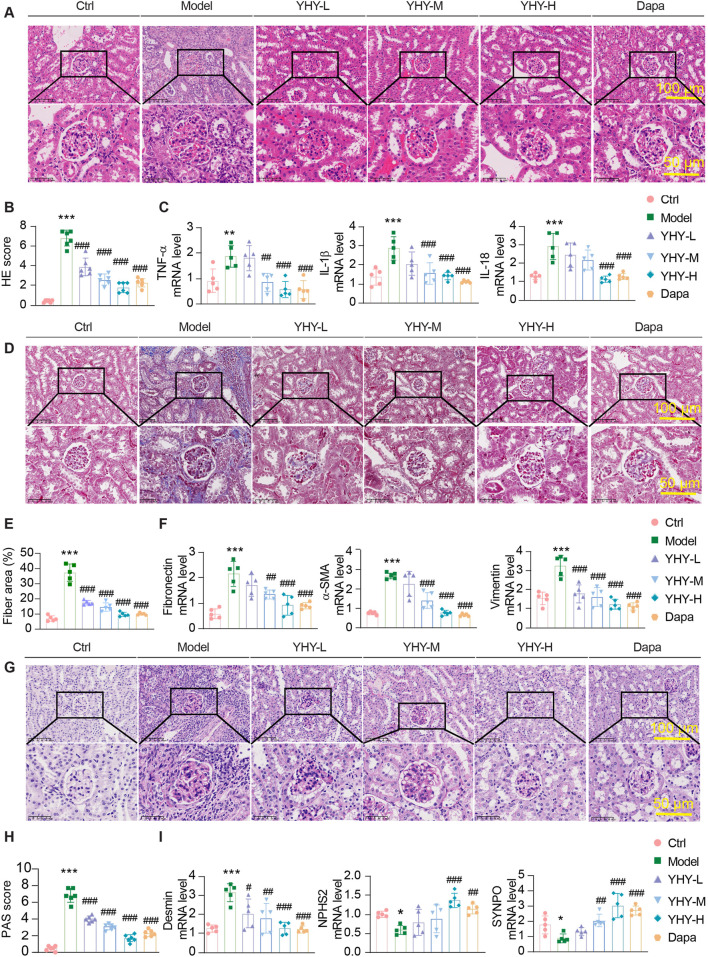
Effects of YHY on Renal Pathological Morphology, and Glomerular Fibrosis in Mice. **(A,B)** HE and pathological score statistical chart (scale bar = 50 μm and 100 μm). **(D,E)** Masson staining and collagen area fraction (scale bar = 50 μm and 100 μm). **(G,H)** PAS and pathological score statistical chart (scale bar = 50 μm and 100 μm). **(C,F,I)** mRNA expressions of pro-inflammatory cytokines (TNF-α, IL-1β, and IL-18), fibrosis-associated proteins (Fibronectin, α-sma, and Vimentin), and podocyte injury markers (Desmin, NPHS2, and SYNPO). Data were shown as mean ± SD, ^*^ *P* < 0.05 and ^**^ *P* < 0.01, ^***^ *P* < 0.001 vs. Ctrl; ^#^ *P* < 0.05, ^##^ *P* < 0.01, ^###^ *P* < 0.001 vs. Model.

A significant corpus of literature has established that DN is marked by a range of renal pathological changes, including glomerular hypertrophy, inflammatory cell infiltration, mesangial matrix expansion, glycogen accumulation, and capillary dilation (Dwivedi and Sikarwar, 2025). Histopathological analysis with HE staining (Hematoxylin-Eosin staining) demonstrated inflammatory cell infiltration and structural damage in the renal tissues of diabetes model mice, which was considerably improved by YHY therapy (Figures 3A,B). The expression of pro-inflammatory cytokines (Tumour necrosis factor-α, Interleukin-1β, and Interleukin-18) was markedly increased in diabetic mice but dramatically reduced by medium- and high-dose YHY treatment (Figure 3C). Masson’s trichrome staining demonstrated significant collagen accumulation and glomerular fibrosis in the model group (Figures 3D,E), corroborated by elevated mRNA levels of fibrosis-associated markers (Fibronectin, α-smooth muscle actin, and Vimentin) as measured by qPCR (Figure 3F). PAS examination revealed glycogen accumulation and thickening of the glomerular basement membrane in diabetic mice. Additionally, podocyte damage markers showed elevated Desmin expression, while the expression levels of NPHS2 and Synaptopodin were significantly decreased. These changes were significantly reversed after YHY intervention (Figures 3G–I). Taken together, these data collectively indicate that YHY therapy reduces renal pathology in DN by diminishing inflammatory responses, fibrosis development, and podocyte injury, therefore maintaining kidney structural integrity.

### 3.4 YHY attenuates renal dysfunction by regulating autophagy-mediated by mTOR

**FIGURE 4:**
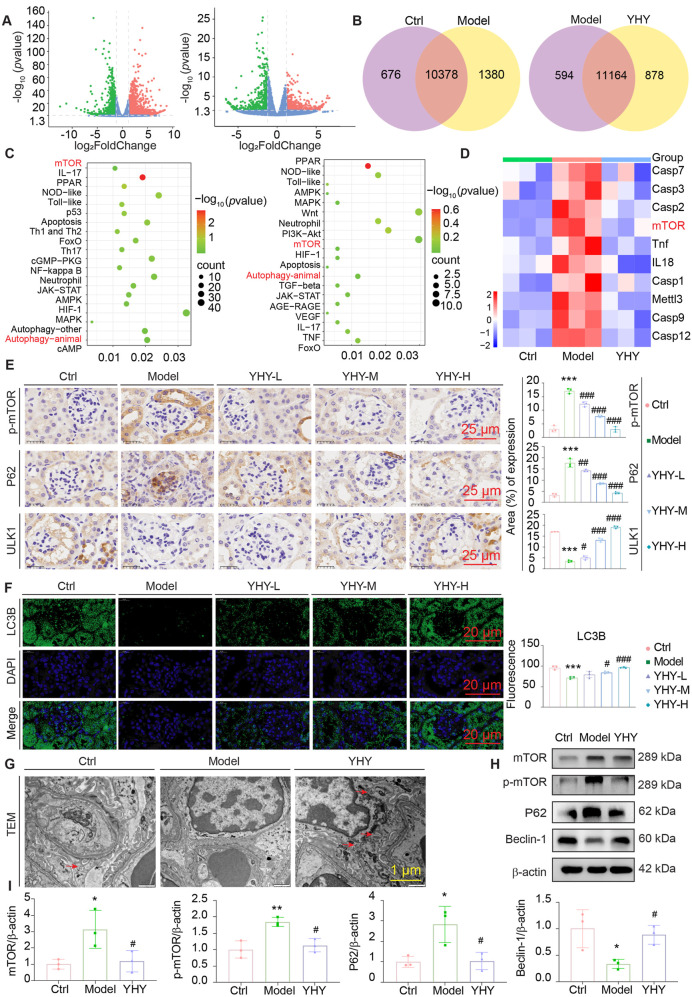
Transcriptomic-related analysis and Effects of YHY on the expressions of autophagy-related substances in the kidneys of *db/db* mice. **(A)** Volcano diagrams of Ctrl vs. Model and Model vs. YHY. **(B)** Venn diagram of Ctrl vs. Model and Model vs. YHY. **(C)** KEGG graph of Ctrl vs. Model and Model vs. YHY. **(D)** Heat maps of differentially expressed genes in the Ctrl, Model and YHY. **(E)** Immunohistochemical representative image of p-mTOR, P62, ULK1 and its statistical graphs (scale bar = 25 μm). **(F)** Immunofluorescence representative images of LC3B and its statistical graphs (scale bar = 20 μm). **(G)** Transmission electron microscopy (TEM): observe autophagic vesicles. **(H,I)** Western blot analysis of mTOR, p-mTOR, P62, Beclin-1 protein expression in tissues and its statistical graphs. Data were shown as mean ± SD, ^*^ *P* < 0.05 and ^**^ *P* < 0.01, ^***^ *P* < 0.001 vs. Ctrl; ^#^ *P* < 0.05, ^##^ *P* < 0.01, ^###^ *P* < 0.001 vs. Model.

To clarify the molecular processes responsible for the protective effects of YHY on DN, transcriptome profiling was conducted. Integrative transcriptome and functional analysis revealed mTOR-mediated autophagic dysregulation in DN and its specific mitigation by YHY. A comparative study identified 2,644 genes that were substantially upregulated and 1,368 genes that were downregulated in the Model group relative to the Ctrl group. The YHY intervention significantly rectified this transcriptional imbalance, leading to the overexpression of 550 genes and the downregulation of 515 genes compared to the Model group (Figures 4A,B). KEGG pathway enrichment analysis revealed multiple markedly modified signalling pathways, notably the mTOR signalling pathway and autophagy-related activities, both of which play a crucial role in the pathogenesis of diabetic renal damage. Autophagy impairment has been associated with podocyte dysfunction and the advancement of renal injury. Transcriptomic results suggest that YHY may exert its therapeutic benefits by regulating mTOR signalling and autophagy-related pathways (Figures 4C,D). Additional validation in renal tissues of diabetic mice revealed considerable dysregulation of autophagy-related proteins, evidenced by heightened levels of phosphorylated mTOR (p-mTOR) and increased accumulation of Sequestosome-1 (P62), and diminished expression of UNC-51-like kinase 1 (ULK1) (Figure 4E). The modifications were dose-dependently improved after YHY therapy. In accordance with these findings, immunofluorescence analysis demonstrated a significant decrease in Microtubule-Associated Protein 1 Light Chain 3B (LC3B) puncta in the Model group, which was subsequently recovered with YHY administration. Ultrastructural analysis via transmission electron microscopy (TEM) confirmed that diabetic podocytes displayed marked thickening of the glomerular basement membrane and a substantial reduction in autophagosome formation-pathological characteristics that were mitigated by YHY treatment (Figures 4F,G). To clarify the molecular processes behind the renoprotective benefits of YHY, we evaluated critical indicators of autophagy signalling in renal tissues. Western blot analysis demonstrated significant dysregulation of autophagy-related proteins in diabetic renal tissues, with the model group showing markedly diminished Beclin-1 expression, increased P62 accumulation, and an elevated phosphorylated mTOR to total mTOR ratio (Phospho-mTOR/mTOR) relative to normal controls (Figures 4H,I). Altered mRNA expression of autophagy-related genes (P62, Beclin-1, and mTOR) in the renal tissues of *db/db* mice was observed via qPCR (Supplementary Figure 2A). YHY treatment effectively normalised these pathological changes, exhibiting a dose-dependent restoration of Beclin-1 levels, a decrease in P62 accumulation, and modulation of mTOR phosphorylation status. This collectively indicates that the therapeutic effects of YHY in alleviating DN are mediated through the regulation of autophagy via the mTOR-dependent pathway.

### 3.5 YHY improves high glucose-induced renal podocyte injury

**FIGURE 5:**
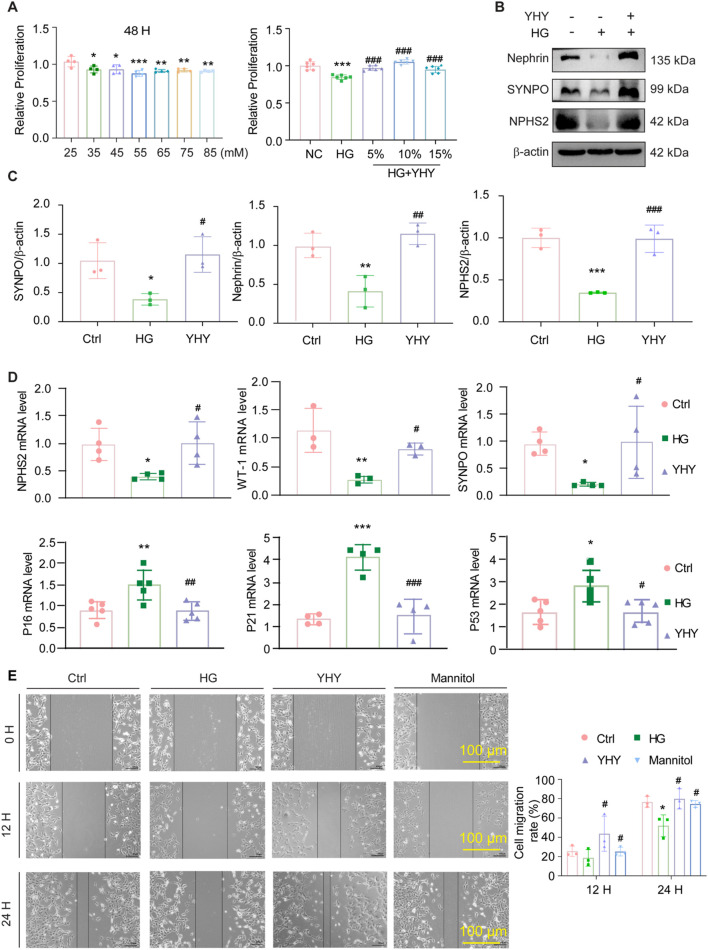
YHY’s renoprotective effects in mitigating HG-induced podocyte dysfunction. **(A)** OD value statistical chart of CCK8: modeling time, modeling concentration and the proportion of YHY drug-containing serum for screening MPC-5 cells. **(B,C)** Western blot analysis of Nephrin, SYNPO, NPHS2 protein expression in cells and its statistical graphs. **(D)** mRNA expressions of podocyte injury markers (Nephrin, WT-1 and SYNPO), and senescence markers (P16, P21, P53) in cells. **(E)** 12-Hour and 24-Hour wound healing assays and representative images from the scratch test and its statistical graph (scale bar = 100 μm). Data were shown as mean ± SD, ^*^ *P* < 0.05 and ^**^ *P* < 0.01, ^***^ *P* < 0.001 vs. Ctrl; ^#^ *P* < 0.05, ^##^ *P* < 0.01, ^###^ *P* < 0.001 vs. Model.

Considering the critical significance of podocyte damage in the advancement of DN, we concentrated on clarifying the protective effects of YHY on renal podocytes *in vitro* (Supplementary Figure 2B). MPC-5 podocytes were subjected to a hyperglycaemic milieu by exposure to high glucose for 48 Hours, resulting in a significant decrease in cell viability and the induction of cytotoxic effects (Figure 5A). In accordance with this, Western blot analysis demonstrated reduced expression of essential podocyte markers (Nephrin, Synaptopodin [SYNPO], and NPHS2), corroborating HG-induced podocyte damage (Figures 5B,C). The qPCR further indicated the downregulation of Wilms tumor 1 protein (WT-1), SYNPO, and NPHS2 mRNA, alongside dysregulated senescence-related markers (P21, P53, and P16) (Figure 5D). Treatment with YHY-containing serum markedly reinstated cell viability and rectified these changes in both protein and gene expression. Furthermore, scratch wound experiments demonstrated significantly diminished migration in HG-treated podocytes, evidenced by decreased wound closure at 12 and 24 Hours (Figure 5E). In contrast, YHY intervention substantially reinstated migratory capacity, highlighting its protective action against HG-induced podocyte dysfunction.

### 3.6 YHY improves high glucose-induced renal podocyte injury by activating autophagy-mediated by mTOR

**FIGURE 6:**
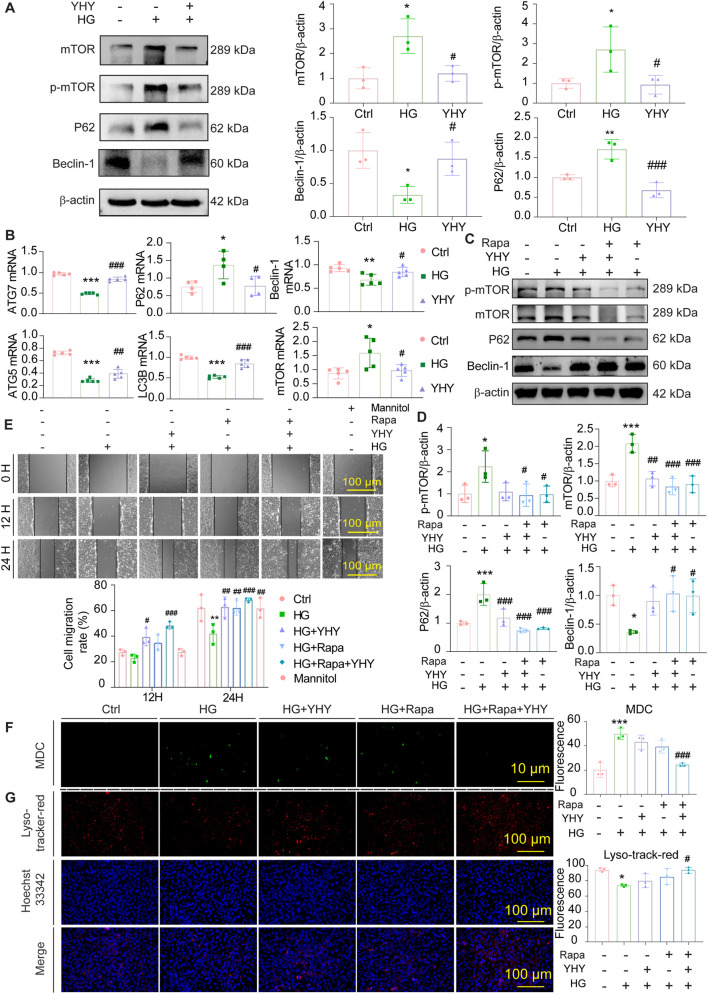
YHY exerts its podocyte-protective effects under high-glucose conditions through mTOR-dependent modulation of autophagic activity. **(A)** Western blot analysis of mTOR, p-mTOR, P62 and Beclin-1 protein expression in cells and its statistical graphs. **(B)** mRNA expressions of autophagy-related genes in cells (ATG5, ATG7, P62, Beclin-1, LC3B, and mTOR). **(C,D)** Western blot analysis of mTOR, p-mTOR, P62 and Beclin-1 protein expression in cells under rapamycin intervention and its statistical graphs. **(E)** 12-Hour and 24-Hour wound healing assays and representative images from the scratch test under rapamycin intervention and its statistical graph (scale bar = 100 μm). **(F)** MDC staining and its statistical graph (scale bar = 10 μm). **(G)** Lyso-Tracker Red/Hoechst 33342 staining and its statistical graph (scale bar = 100 μm). Data were shown as mean ± SD, ^*^ *P* < 0.05 and ^**^ *P* < 0.01, ^***^ *P* < 0.001 vs. Ctrl; ^#^ *P* < 0.05, ^##^ *P* < 0.01, ^###^ *P* < 0.001 vs. Model.

To clarify the protective mechanisms of YHY against podocyte injury generated by high glucose, we concentrated on mTOR-regulated autophagy pathways. Molecular and functional analysis demonstrated that YHY reinstates autophagy in podocytes disrupted by high glucose through mTOR signalling, with enhanced efficiency noted when combined with mTOR inhibition. Under raised glucose circumstances, MPC-5 podocytes demonstrated severe disruption of autophagic flow, as seen by Western blot analysis showing markedly reduced Beclin-1 expression, increased P62 accumulation, and heightened mTOR phosphorylation compared to normal glucose (Figure 6A). The data were corroborated by qPCR, which revealed transcriptional downregulation of autophagy-related genes (Beclin-1, LC3B, autophagy related 5 [ATG5], autophagy related 7 [ATG7]) and overexpression of mTOR and P62. The YHY therapy successfully restored the expression of these autophagy markers at both protein and mRNA levels (Figure 6B). Supplementary investigations utilising rapamycin, a mTOR inhibitor (Supplementary Figure 2C), further substantiated the participation of this pathway, demonstrating a diminished p-mTOR/mTOR ratio, decreased P62 levels, and elevated Beclin-1 (Figures 6C,D). The concurrent administration of YHY and rapamycin resulted in a synergistic reduction of abnormal mRNA expression in podocyte damage indicators (WT-1, SYNPO) and autophagy-related genes (P62, ATG5, ATG7) (Supplementary Figure 2D). Functional scratch experiments demonstrated that both YHY and rapamycin reinstated podocyte migration, with wound closure rates reverting to baseline levels at 12 and 24 h (Figure 6E). Lysosomal integrity and autophagic vesicle production, evaluated using Lyso-Tracker Red/Hoechst and MDC staining, were enhanced by both YHY and rapamycin, with the combination producing the most significant restoration of autophagic function (Figures 6F,G). These findings jointly demonstrate that YHY mitigates HG-induced podocyte damage predominantly via mTOR-dependent autophagy repair.

### 3.7 YHY attenuates podocyte injury in DN by targeting METTL3 to regulate m6A-dependent mTOR autophagy signalling

**FIGURE 7:**
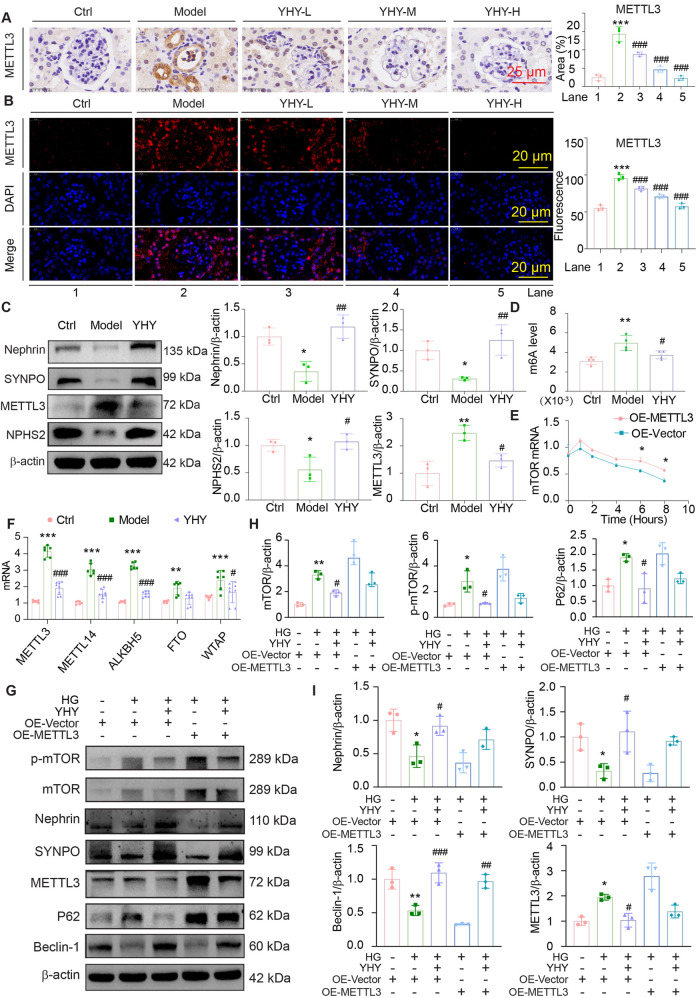
YHY exerts therapeutic effects by modulating this METTL3-m6A-mTOR regulatory axis. **(A)** Immunohistochemical representative image of METTL3 and its statistical graph (scale bar = 25 μm). **(B)** Immunofluorescence representative images of LC3B and its statistical graphs (scale bar = 20 μm). **(C)** Western blot analysis of Nephrin, SYNPO, NPHS2 and METTL3 protein expression in tissues and its statistical graphs. **(D)** Quantitative evaluation statistical chart of m6A RNA methylation in tissues. **(E)** Statistical chart of mTOR mRNA stability. **(F)** mRNA expressions of m6A-related genes (METTL3, METTL14, ALKBH5, FTO and WTAP). **(G–I)** Western blot analysis of mTOR, p-mTOR, Nephrin, SYNPO, METTL3, P62 and Beclin-1 protein expression in cells when METTL3 is overexpressed and its statistical graphs. Data were shown as mean ± SD, ^*^ *P* < 0.05 and ^**^ *P* < 0.01, ^***^ *P* < 0.001 vs. Ctrl; ^#^ *P* < 0.05, ^##^ *P* < 0.01, ^###^ *P* < 0.001 vs. Model.

**FIGURE 8:**
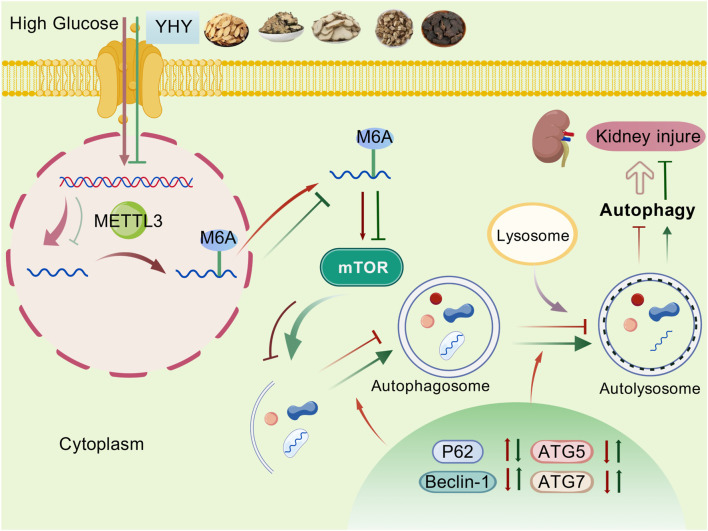
Schematic diagram of the mechanism by which YHY regulates autophagy through the METTL3-m6A-mTOR axis to improve hyperglycemic kidney injury.

Further, this study analyzed transcriptome datasets from the GEO database (accession numbers: GSE96804 and GSE30529) to identify principal regulators associated with DN (Supplementary Figure 3). Our study indicated a significant elevation of METTL3 in DN samples, which garnered our interest due to its pivotal role in epigenetic regulation (Supplementary Figure 4A, B). METTL3, a prominent methyltransferase, is associated with the regulation of mRNA stability and translation efficiency, notably in relation to mTOR, a key regulator of autophagy and cellular homeostasis. Molecular docking analysis revealed potential interactions between multiple detected metabolites of YHY and the catalytic site of METTL3 (Supplementary Figure 4C), suggesting a plausible mechanism whereby YHY may modulate METTL3 activity. Immunohistochemical and immunofluorescence staining demonstrated that kidney METTL3 expression in diabetic mice was significantly elevated compared to normal controls, an effect that was reversed by YHY administration in a dose-dependent manner (Figures 7A,B). Western blot and qPCR analysis corroborated the downregulation of podocyte markers (Nephrin, SYNPO, and NPHS2), while global m6A levels were increased in diabetic kidneys, with METTL3 specifically upregulated among m6A-modifying enzymes (Figures 7C,D,F; Supplementary Figure 2A). In high glucose-stimulated MPC-5 podocytes, knockdown of METTL3 downregulated mTOR expression and suppressed autophagy signaling (Supplementary Figure 5A), whereas METTL3 overexpression exacerbated cellular injury and autophagy dysfunction. These adverse effects were characterized by mTOR pathway hyperactivation (elevated p-mTOR/mTOR ratio), consequent autophagic flux blockade (increased P62 accumulation alongside reduced Beclin-1 and LC3B expression), and severe podocyte injury (decreased nephrin, SYNPO, and NPHS2 with concurrent Desmin upregulation). Notably, YHY treatment relieved this pathogenic cascade (Figures 7G–I; Supplementary Figures 5B, C). Mechanistically, METTL3 increased the stability of mTOR mRNA in a time-dependent manner (Figure 7E). These findings collectively indicate that YHY alleviates podocyte damage and restores autophagy in DN by regulating the METTL3-m6A-mTOR pathway (Figure 8).

## 4 Discussion

Recent data highlights the critical importance of podocyte autophagy dysregulation in the course of DN, since this conserved cellular mechanism preserves the integrity of the glomerular filtration barrier by eliminating damaged organelles and proteins (Teh et al., 2022). Elevated glucose levels hinder autophagic flow and worsen podocyte damage (Cao et al., 2024). *In vivo* studies indicated that YHY therapy not only lowered fasting blood glucose but also markedly diminished glycated hemoglobin levels (Figure 2), indicating its potential for prolonged glycemic regulation. In the early and middle stages of the experiment, diabetic mice were in a serious “wasting state”, and energy was effectively utilized in the late stage of drug treatment. *Db/db* mice in the YHY-H group showed a faster body weight gain compared with other groups, potentially due to their increased food intake (Figure 2H). Simultaneously, YHY enhanced renal function and significantly improved glomerular histology (Figure 3). This is similar to the findings of Shen et al. (2024) about Yiqi Huoxue Yangyin Qingre Formula on autophagy signaling pathway in renal tissue of DN. These data jointly demonstrate YHY’s dual therapeutic potential in concurrently treating hyperglycemia and the advancement of DN.

The mechanistic target of mTOR, a key nutrient sensor and catalytic core of mTORC1/2, critically regulates autophagy in DN. Its hyperactivation has been mechanistically associated with podocyte damage and failure of the glomerular filtration barrier, identifying this pathway as a crucial modulator of autophagic dysregulation in DN. Herein, transcriptomic analysis demonstrated YHY’s ability to simultaneously influence critical pathways, including inflammatory (IL-17/TNF/NOD-like/Toll-like receptor), stress response (p53/TGF-β), and metabolic (mTOR-autophagy) networks, with the mTOR pathway identified as a pivotal component of its mechanism of action. In *db/db* mice, YHY treatment normalized mTOR phosphorylation and restored autophagic flux (Figure 4). The therapeutic equivalence of YHY and rapamycin in autophagy activation was further substantiated in HG-stimulated MPC-5 podocytes, where combination treatment exhibited synergistic effects in mitigating podocyte injury (Figures 5, 6). This mechanism is similar to the research results of Lai W et al. (2023), which concluded that restoring podocyte autophagy is essential for discovering new pharmacological interventions for DN. These findings jointly establish YHY as a prospective therapeutic candidate for DN by its coordinated modulation of mTOR-dependent autophagy and maintenance of podocyte structural integrity. However, the current assessment of autophagic flux relied on static protein markers, which, while informative, do not fully capture dynamic autophagic activity. Future studies employing lysosomal inhibitors are warranted to comprehensively evaluate the impact of YHY on autophagic flux and definitively confirm its mechanism of action.

Accumulating evidence indicates that METTL3 post-transcriptionally regulates autophagy by affecting the stability or expression of autophagy-related genes. For example, METTL3 modulates autophagic activity in ischemic cardiomyocytes and endometrial cells via ATG7/P62 (Li X. et al., 2025; Zhang Y. et al., 2025). Moreover, METTL3 overexpression not only reinforces the PI3K/AKT/mTOR axis to suppress autophagy and promote protein synthesis but also introducing aberrant m6A modifications in transcripts such as MDM2 (Wu et al., 2025), TIMP2 (Jiang et al., 2022), and TRIM2 (Xu et al., 2025), thereby aggravating podocyte injury in DN. In this study, integrated analysis of clinical transcriptome datasets (GSE96804, GSE30529) identified METTL3 as significantly upregulated in DN, suggesting its pathogenic role. In addition, molecular docking revealed high binding affinity between YHY’s detected metabolites and METTL3, supporting its therapeutic relevance. Consistent with these predictions, our RNA-seq data confirmed markedly elevated METTL3 expression in renal tissues of *db/db* mice compared to the control group, indicating epitranscriptomic dysregulation in DN. Conversely, YHY treatment effectively suppressed METTL3 expression, which correlated with improved renal histopathology and function, suggesting that METTL3 inhibition contributes to DN amelioration. The aforementioned result was further supported by *in vitro* studies utilizing high glucose-stimulated MPC-5 podocytes, where METTL3 overexpression exacerbated cellular injury and suppressed autophagy through activation of the mTOR pathway-effects that were significantly reversed by YHY administration (Figure 7). Functionally, METTL3 knockdown phenocopied the protective effect of YHY treatment by activating autophagic activity (Supplementary Figure 5A). These results demonstrate that YHY alleviates podocyte injury and restores autophagy by suppressing METTL3-dependent m6A methylation and downstream mTOR signaling (Figure 8). However, the precise role of m6A methylation in DN, particularly its direct regulation of mTOR mRNA stability, remains to be elucidated. Future studies integrating approaches such as meRIP-seq and RIP-qPCR with METTL3 perturbation models will be essential to delineate this epitranscriptomic mechanism.

Our UPLC-MS/MS analysis successfully identified multiple chemical metabolites within the formula. It is worth noting that these metabolites have been independently reported to possess biological activities relevant to our findings. For instance, Puerarin exerts anti-hepatocellular carcinoma effects by suppressing glycolysis via the FBXW7/mTOR axis (Cai et al., 2023). In the context of DN, Puerarin also modulates the PERK/eIF2α/ATF4 pathway to affect autophagy and renal function (Xu et al., 2020). Similarly, Emodin induces apoptosis and ferroptosis in gastric cancer cells by targeting mTOR signaling (Xi et al., 2024). Furthermore, Genistein, in conjunction with Myd88, activates autophagy in high glucose-induced renal podocytes (Wang et al., 2018). Astragaloside IV demonstrates protective effects by alleviating podocyte apoptosis via the TXNIP/NLRP3/GSDMD pathway in DN (Hu et al., 2024) and, when administered intravenously, promotes METTL3-mediated m6A modification of SIRT1 (Liu et al., 2025). Although the precise contribution of individual metabolites to the observed pharmacological effects and their direct interaction with the METTL3/m6A/mTOR pathway requires further investigation through activity-guided fractionation and targeted studies, it is highly plausible that the integrated action of these metabolites in YHY collectively contributes to the observed suppression of METTL3-mediated m6A methylation, leading to mTOR inhibition, autophagy activation, and ultimately, podocyte protection. This ‘multi-metabolite, multi-target’ action mode is a hallmark of TCM formulas. Furthermore, a recent study by Zhang Y. et al. (2025) demonstrated that wogonoside, a flavonoid from Scutellaria baicalensis, alleviates podocyte injury by inhibiting the TLR4/NF-κB inflammatory pathway. Together, these findings highlight the multifaceted therapeutic potential of natural products, whether as single compounds or complex formulas, in targeting distinct pathogenic pathways in DN, and suggest that future exploration of synergistic combinations may yield additional therapeutic benefits.

Besides, a balanced evaluation of YHY against conventional therapeutic regimens is essential to delineate its relative advantages and limitations, thereby clarifying its translational value for DN management. Dapagliflozin lowers blood glucose through insulin-independent mechanisms involving inhibition of renal tubular glucose reabsorption and promotion of urinary glucose excretion, achieving both antihypertensive and renoprotective effects. However, this agent is associated with adverse effects including polyuria, polydipsia, weight loss, and decreased appetite. In contrast, the Chinese botanical drug compound YHY demonstrates a favorable safety profile while exerting multifactorial therapeutic effects in DN. Our experimental data reveal that YHY administration not only ameliorates hyperglycemia and proteinuria in *db/db* mice but also preserves renal structural integrity and functional capacity, underscoring its dual role in glycemic control and nephroprotection. Although YHY exhibits the characteristically gradual onset of action common to TCM formulations, its therapeutic effects against DN prove comparable to dapagliflozin. Particularly noteworthy is the observed ability of YHY to promote appropriate weight gain during later treatment phases, which may effectively counterbalance dapagliflozin-induced weight loss. This complementary relationship suggests that combination therapy could yield synergistic benefits, maintain effective glycemic control and renal protection while promote more stable metabolic homeostasis and body weight. However, the translational relevance of our findings is tempered by the inherent limitations of the *db/db* mouse model. Future studies should incorporate complementary pre-clinical models and patient-derived podocytes, alongside more comprehensive functional endpoints and appropriate osmotic controls. Furthermore, comprehensive pharmacokinetic profiling and toxicological assessment of YHY are warranted. Such investigations would strengthen the translational relevance of YHY and support its development as a multi-target therapeutic strategy for patients with DN.

## 5 Conclusion

In conclusion, this study demonstrates that YHY, a multi-botanical drug TCM formula, significantly alleviates DN by mitigating hyperglycemia, podocyte injury, and renal dysfunction. Combining network pharmacology, transcriptomics, and experimental validation, this study revealed that YHY’s renoprotective effect and its mechanism driven by the regulation of METTL3-dependent m6A methylation to suppress mTOR and activate autophagy. Our findings elucidate both YHY’s novel epigenetic dimension and its underlying chemical and mechanistic basis, establishing a scientific foundation for its clinical development as a prospective DN therapeutic agent.

## Data Availability

The datasets presented in this study can be found in online repositories. The names of the repository/repositories and accession number(s) can be found in the article/Supplementary Material.

## Glossary

ACE: Angiotensin converting enzyme
APS: Astragalus polysaccharide
ARB: Angiotensin receptor blocker
ATG5: autophagy related 5
ATG7: autophagy related 7
Beclin-1: BECN1
Dapa: Dapagliflozin
DM: diabetes mellitus
DN: Diabetic nephropathy
FBG: Fasting blood glucose
Fn: Fibronectin
GLP-1: Glucagon-like peptide-1
HbA1c: Glycosylated hemoglobin, type A1C
HE: Hematoxylin and Eosin Staining
HG: High-glucose
LC3B: Microtubule-associated protein 1A/1B-light chain 3B
m6A: N6-methyladenosine
MASSON: Masson’s Trichrome Staining
METTL3: Methyltransferase like 3
METTL14: Methyltransferase like 14
mTOR: Mammalian target of rapamycin
P16: Multiple tumor suppressor 1
P21: Cyclin-Dependent Kinase Inhibitor 1A (CDKN1A)
P53: Tumor Protein p53
P62: Sequestosome 1 (SQSTM1)
PAS: Periodic Acid-Schiff
p-mTOR: Phosphorylated mammalian target of rapamycin
SGLT2: Sodium-glucose cotransporter 2
SYNPO: Synaptopodin
TCM: Traditional chinese medicine
TEM: Transmission electron microscopy
ULK1: Unc-51 Like Autophagy Activating Kinase 1
WT-1: Wilms tumor 1 protein
YHY: Yiqi Huoxue Yangyin Decoction
YHY-H: Yiqi Huoxue Yangyin Decoction High-dose group
YHY-L: Yiqi Huoxue Yangyin Decoction Low-dose group
YHY-M: Yiqi Huoxue Yangyin Decoction Medium-dose group
