# Supplementary material for: Yiqi Huoxue Yangyin Decoction attenuates diabetic nephropathy in db/db mice by modulating METTL3-mediated m6A methylation of mTOR to restore podocyte autophagy
Source: Front Pharmacol. 2026 Apr 7;17:1783423. doi: 10.3389/fphar.2026.1783423 (PMC13095182; doi:10.3389/fphar.2026.1783423)
Supplement: Supplementary file 1 [file Supplementaryfile1.docx]

Supplementary Material

# Supplementary Figures and Tables

## Supplementary Tables

**Table S1. Primer sequences used for qPCR analysis**

| **Gene** | **Forward primer** | **Reverse primer** |
| --- | --- | --- |
| β-actin | GTGACGTTGACATCCGTAAAGA | GCCGGACTCATCGTACTCC |
| Beclin-1 | TCGGCTTCGTTCTTCCACCTC | GTCACCATTGTCCTCAAAGTCCTC |
| LC3B | GCGGGTGATTATAGAGCGATACAAG | CGCCGTCTGATTATCTTGATGAGC |
| ATG5 | AAGCTGTTCCGGCCTGTGG | ATCATCACCTGGCTCCTCTTCTC |
| ATG7 | AGCAGTGATGACCGCATGAATG | CCAGGCTGACAGGAAGAACATTATC |
| P53 | TGAACCGCCGACCTATCCTTAC | TCCCAGGGCAGGCACAAAC |
| P21 | CCGTGGACAGTGAGCAGTTG | CCTCCAGCGGCGTCTCC |
| SYNPO | GTGAGGAAGAGGAAGTGCCATTG | GGGAGGGTTTGGTGAGGACTG |
| NPHS2 | CCTGAGGATGGCGGCTGAG | AGTGGCTGGCTTCTCTGTGG |
| P62 | CATTGAGGTTGACATTGATGTGGAAC | GTTACTCTTGTCTTCTGTGCCTGTG |
| P16 | CCGATTCAGGTGATGATGATGGG | CGGGCGGGAGAAGGTAGTG |
| Desmin | GCTAAGAACATCTCTGAGGCTGAAG | CATCATCTCCTGCTTGGCTTGG |
| WT-1 | CCTGTCGCTACGGACCCTTC | CGGATGGTAGGCTGGCTCTC |

**Table S1. Primer sequences used for qPCR analysis (Continued Table)**

| **Gene** | **Forward primer** | **Reverse primer** |
| --- | --- | --- |
| METTL3 | TCCATCCGTCTTGCCATCTCTAC | CCTCGCTTTACCTCAATCAACTCC |
| ALKBH5 | CCGCCGCTGAGCCTTACC | CTCTTCCTCCTTCTGCAACTGATG |
| FTO | *GGAGGAACGAGAGCGGGAAG* | *GCTGCCACTGCTGATAGAACTC* |
| WATP | GCAAGTTCAGCAGCCGAGTG | TCTTTAGTCTGTTCCAGTTCACCTTTC |
| METTL14 | AGACATAGAAGCCTTTGACATCAGAG | TCCAGCATTTCTCATTCGCAGTG |
| mTOR | GATGTGCCGAGACCTTGAGTTG | GCCTCTGCTTGGATGTGATGAC |
| IL-1β | GACCTTCCAGGATGAGGACA | AGCTCATATGGGTCCGACAG |
| TNF-α | CGTCGTAGCAAACCACCAAG | TTGAAGAGAACCTGGGAGTAGACA |
| IL-18 | AGACTACTTCCTGAGCACAAGA | TGTCCTTACCAATGGTTCTCACT |
| α-SMA | ACTGGGACGACATGGAAAAG | GTTCAGTGGTGCCTCTGTCA |
| Fibronectin | ATGTGGACCCCTCCTGATAGT | GCCCAGTGATTTCAGCAAAGG |
| Vimentin | TATCCTGGTTGTTAATTGCGACCG | GGACTGGCACAAATGACATGCTTAG |

**Table S2. Antibodies used for WB analysis**

| **Antibodies** | **Item number** | **Dilution** | **Company and location** |
| --- | --- | --- | --- |
| mTOR | A11355 | 1:500 for WB | Abclonal, China |
| p-mTOR | AP0115 | 1:500 for WB | Abclonal, China |
| NPHS2 | 20384-1-AP | 1:500 for WB | Proteintech, China |
| SYNPO | 21064-1-AP | 1:2000 for WB | Proteintech, China |
| METTL3 | A19079 | 1:1000 for WB | Abclonal, China |
| P62 | 84826-1-RR | 1:5000 for WB | Proteintech, China |
| Beclin-1 | A21191 | 1:2000 for WB | Abclonal, China |
| Nephrin | HA722886 | 1:1000 for WB | Huabio, China |
| β-actin | EM21002 | 1:10000 for WB | Huabio, China |

| **Table S3. The main ingredient list of YHY** | | | | | | |
| --- | --- | --- | --- | --- | --- | --- |
| **NO.** | **RT**  **(min)** | **Component name** | **Formula** | **Mass error (ppm)** | **Response** | **Origin** |
| 1 | 0.78 | L-Canavanine | C_5_H_12_N_4_O_3_ | -2.3 | 47034 | Huang-Qi |
| 2 | 0.8 | L-Arginine | C_6_H_14_N_4_O_2_ | -1.8 | 16390 | Huang-Qi |
| 3 | 0.81 | Verbascose | C_30_H_52_O_26_ | -0.2 | 134621 | Shen-Di-Huang |
| 4 | 0.89 | Proline | C_5_H_9_NO_2_ | 0.8 | 146425 | Huang-Qi |
| 5 | 1.37 | DL-Pyroglutamic acid | C_5_H_7_NO_3_ | -0.5 | 73338 | Shen-Di-Huang |
| 6 | 1.49 | Danmelittoside | C_15_H_22_O_10_ | -0.3 | 251339 | Shen-Di-Huang |
| 7 | 1.49 | p-Coumaric acid | C_9_H_8_O_3_ | -1 | 10564 | Huang-Qi |
| 8 | 1.55 | 5-Hydroxymethyl-2- furancarboxylic acid | C_6_H_6_O_4_ | -2 | 74243 | Shen-Di-Huang |
| 9 | 2.5 | Adenine | C_5_H_5_N_5_ | -1.4 | 39095 | Huang-Qi |
| 10 | 4.51 | Calycosin-7-0-beta-D glucuronide | C_22_H_20_O_11_ | -1.2 | 5098 | Huang-Qi |
| 11 | 4.98 | 3-Cyclohexen-1-ol | C_6_H_10_O | -1.4 | 498250 | Chuan-Xiong |
| 12 | 5.12 | Adipic acid | C_6_H_10_O_4_ | -1.7 | 49424 | Chuan-Xiong |
| 13 | 5.22 | Caproic acid | C_6_H_12_O_2_ | -0.3 | 83851 | Shen-Di-Huang |
| 14 | 5.59 | Puerarin 4'-O-glucoside | C_27_H_30_O_14_ | 0.1 | 2307043 | Ge-Gen |
| 15 | 6.09 | Daidzin | C_21_H_20_O_9_ | -0.4 | 610502 | Ge-Gen |
| **Table S3. The main ingredient list of YHY (Continued Table)** | | | | | | |
| **NO.** | **RT**  **(min)** | **Component name** | **Formula** | **Mass error (ppm)** | **Response** | **Origin** |
| 16 | 6.09 | 4',6,7-Trihydroxyisoflavone-6-methylether-7-O-beta-D-xylopyranosyl-(1->6)-beta-D-glucopyranoside | C_27_H_30_O_14_ | 0.4 | 774430 | Ge-Gen |
| 17 | 6.29 | 3'-Hydroxy puerarin | C_21_H_20_O_10_ | 0.3 | 2916740 | Ge-Gen |
| 18 | 6.29 | Riboflavin | C_17_H_20_N_4_O_6_ | 2.7 | 277708 | Huang-Qi |
| 19 | 7.49 | Purpureaside C | C_35_H_46_O_20_ | -0.5 | 227663 | Shen-Di-Huang |
| 20 | 7.71 | Chrysophanol | C_15_H_10_O_4_ | -1.6 | 118838 | Shen-Di-Huang |
| 21 | 7.92 | 8,8'-Difenulic acid | C_20_H_18_O_8_ | 0.1 | 15510 | Chuan-Xiong |
| 22 | 8.01 | Daidzin | C_21_H_20_O_9_ | -0.7 | 1402776 | Ge-Gen |
| 23 | 8.01 | Rhamnocitrin 3-O-glucoside | C_22_H_22_O_11_ | -0.3 | 940795 | Huang-Qi |
| 24 | 8.49 | Kuzubutenolide A | C_23_H_24_O_10_ | -1.4 | 389077 | Ge-Gen |
| 25 | 8.49 | Pueroside A | C_29_H_34_O_14_ | 1 | 1726787 | Ge-Gen |
| 26 | 8.78 | Kuzubutenolide A | C_23_H_24_O_10_ | 0.1 | 10811 | Ge-Gen |
| 27 | 8.88 | Rehmaionoside A | C_19_H_34_O_8_ | -0.2 | 224387 | Shen-Di-Huang |
| 28 | 9.1 | Pueroside B | C_30_H_36_O_15_ | 2 | 277444 | Ge-Gen |
|  | | | | | | |
| **Table S3. The main ingredient list of YHY (Continued Table)** | | | | | | |
| **NO.** | **RT**  **(min)** | **Component name** | **Formula** | **Mass error (ppm)** | **Response** | **Origin** |
| 29 | 9.15 | 1-Methoxy-2-methylanthraquinone | C_16_H_12_O_3_ | -1 | 202535 | Chuan-Xiong |
| 30 | 9.22 | 4-0-Methylpuerarin | C_22_H_22_O_9_ | -3 | 24006 | Ge-Gen |
| 31 | 9.34 | Genistein | C_15_H_10_O_5_ | -1.7 | 979021 | Ge-GenHuang-Qi |
| 32 | 9.34 | Genistin | C_21_H_20_O_10_ | 0.6 | 221721 | Ge-GenHuang-Qi |
| 33 | 9.51 | Verbascoside | C_29_H_36_O_15_ | -0.1 | 463061 | Huang-Qi |
| 34 | 9.61 | Formononetin 7-O-glucuronide | C_22_H_22_O_9_ | -2 | 24184 | Ge-Gen |
| 35 | 9.7 | 3'-Methoxydaidzein | C_16_H_12_O_5_ | -1.7 | 43023 | Ge-Gen |
| 36 | 9.86 | Senkyunolide F | C_12_H_14_O_3_ | -1.9 | 12770 | Chuan-Xiong |
| 37 | 10.56 | Biochanin A | C₁₆H₁₂O₅ | -1.5 | 32056 | Ge-Gen |
| 38 | 10.66 | Pueroside C | C_24_H_26_O_10_ | -1.1 | 361607 | Ge-Gen |
| 39 | 11.02 | Formononetin | C_16_H_12_O_4_ | -1.1 | 1852956 | Ge-GenHuang-Qi |
| 40 | 11.23 | Methylnissolin | C_17_H_16_O_5_ | -3.4 | 24342 | Huang-Qi |
| 41 | 11.32 | Scoparone | C_11_H_10_O_4_ | -1.2 | 89923 | Ge-Gen |
| 42 | 11.48 | trans Caffeic acid | C_9_H_8_O_4_ | -2.3 | 75117 | Chuan-Xiong |
| **Table S3. The main ingredient list of YHY (Continued Table)** | | | | | | |
| **NO.** | **RT**  **(min)** | **Component name** | **Formula** | **Mass error (ppm)** | **Response** | **Origin** |
| 43 | 11.71 | 9.10-Dimethoxy-pterocarpan-3- 0-β-D-glucopyranoside | C_23_H_26_O_10_ | -1.9 | 294537 | Huang-Qi |
| 44 | 13.04 | SoyasaponinI | C_48_H_78_O_18_ | -3.1 | 76405 | Huang-Qi |
| 45 | 14.15 | AstragalosideIV | C_41_H_68_O_14_ | 1.3 | 13046 | Huang-Qi |
| 46 | 15.64 | Acetylastragaloside | C_47_H_74_O_17_ | 0.5 | 12966 | Huang-Qi |
| 47 | 17.43 | Cucurbitacin B | C_32_H_46_O_8_ | -0.4 | 79565 | Tian-Hua-Fen |
| 48 | 17.94 | Methyl(2E,4E)-hexadeca-2,4-dienoate | C_17_H_30_O_2_ | -2.2 | 106207 | Shen-Di-Huang |
| 49 | 20.34 | Butylphthalide | C_12_H_14_O_2_ | -1.1 | 35747 | Chuan-xiong |
| 50 | 23.94 | (3Z,6S,7R)-3-Butylidene-6-butyryl-7-hydroxy-4,5,6,7-tetrahydroisobenzofuran-1-one | C_16_H_22_O_4_ | -2.5 | 16660 | Chuan-Xiong |

**Table S4. The main ingredient list of YHY drug-containing serum**

| **No.** | **m/z** | **RT(min)** | **q Value** | **Accepted Description** | **Adducts** | **Formula** | **Score** | **Mass Error (ppm)** |
| --- | --- | --- | --- | --- | --- | --- | --- | --- |
| 1 | 223.07 | 1.32 | 0.05 | Naringenin 7-sulfate | M+H-H2O, M+Na | C_8_H_12_N_2_O_4_ | 34.7 | -4.60 |
| 2 | 385.11 | 1.45 | 0.01 | Apigenin | M+Na | C_15_H_22_O_10_ | 40.6 | 1.74 |
| 3 | 627.33 | 5.77 | 0.04 | Glycitein 7-O-glucuronide | M+Na | C_27_H_44_N_10_O_6_ | 39 | 1.31 |
| 4 | 275.02 | 6.45 | 0.01 | Dihydroferulic acid 4-O-sulfate | M-H | C_10_H_12_O_7_S | 40.3 | 2.18 |
| 5 | 253.05 | 6.58 | 0.00 | Daidzein | M-H | C_15_H_10_O_4_ | 42.2 | 2.30 |
| 6 | 417.12 | 6.98 | 0.01 | Glycitein 4'-O-glucuronide | M+H | C_21_H_20_O_9_ | 43.8 | 2.40 |
| 7 | 509.04 | 7.32 | 0.00 | Daidzein 4'-glucuronide-7-sulfate | M-H | C_21_H_18_O_13_S | 36.4 | 2.29 |
| 8 | 255.07 | 7.72 | 0.00 | Dihydrodaidzein | M-H | C_15_H_12_O_4_ | 37.5 | -1.97 |
| 9 | 794.41 | 7.79 | 0.02 | Naringenin | M+Na | C_36_H_62_N_5_O_11_P | 33.7 | 1.03 |
| 10 | 431.10 | 7.84 | 0.01 | Biochanin A | M+H-H2O | C_21_H_20_O_11_ | 43.6 | 1.74 |
| 11 | 461.07 | 7.90 | 0.04 | Kaempferol 3-glucuronide | M-H | C_21_H_18_O_12_ | 37.9 | 1.46 |
|  | | | | | | | | |
|  | | | | | | | | |
| **Table S4. The main ingredient list of YHY drug-containing serum (Continued Table)** | | | | | | | | |
| **No.** | **m/z** | **RT(min)** | **q Value** | **Accepted Description** | **Adducts** | **Formula** | **Score** | **Mass Error (ppm)** |
| 12 | 507.26 | 8.38 | 0.03 | Vitamin A | M+H | C_27_H_38_O_9_ | 35 | -0.51 |
| 13 | 215.00 | 8.41 | 0.01 | 3- Acetylphenol sulfate | M-H | C_8_H_8_O_5_S | 37.8 | -1.46 |
| 14 | 447.09 | 8.64 | 0.00 | naringenin-7-O-glucuronide | M-H | C_21_H_20_O_11_ | 39.2 | 2.20 |
| 15 | 433.12 | 9.54 | 0.00 | 2-O-Caffeoylarbutin | M-H | C_21_H_22_O_10_ | 38.4 | 3.03 |
| 16 | 351.02 | 9.56 | 0.00 | Naringenin 7-sulfate | M-H | C_15_H_12_O_8_S | 42.1 | 2.19 |
| 17 | 957.50 | 9.63 | 0.05 | Cyanidin 3-(6''-dioxalylglucoside) | M+Na | C_46_H_78_O_19_ | 32.5 | -4.03 |
| 18 | 353.03 | 11.81 | 0.00 | Dihydronaringenin-O-sulphate | M-H | C_15_H_14_O_8_S | 41.6 | 1.37 |
| 19 | 455.32 | 14.22 | 0.03 | 5-Hydroxyaloin A | M+H-H2O, M+H | C_29_H_44_O_5_ | 39.7 | 0.00 |
| 20 | 328.25 | 14.94 | 0.03 | Demethoxycurcumin | M+H | C_18_H_33_NO_4_ | 36.5 | -3.26 |
| 21 | 335.24 | 15.29 | 0.04 | Apiforol sulfate | M+H-H2O | C_24_H_32_O_2_ | 41.4 | -0.51 |
| 22 | 527.31 | 16.49 | 0.02 | FMNH2 | M+H | C_31_H_38_N_6_O_2_ | 37.7 | 1.87 |
| 23 | 557.31 | 17.72 | 0.02 | Cucurbitacin B | M-H | C_32_H_46_O_8_ | 33.9 | 3.58 |
|  | | | | | | | | |
| \| **Table S4. The main ingredient list of YHY drug-containing serum (Continued Table)** \| \| --- \| | | | | | | | | |
| **No.** | **m/z** | **RT(min)** | **q Value** | **Accepted Description** | **Adducts** | **Formula** | **Score** | **Mass Error (ppm)** |
| 24 | 345.15 | 18.85 | 0.05 | Apigenin 7-sulfate | M+H | C18H20N2O5 | 39.9 | 2.47 |
| 25 | 109.10 | 23.97 | 0.04 | Daidzein 4'-glucuronide-7-sulfate | M+H-H2O | C8H14O | 46.5 | 3.63 |
| 26 | 203.18 | 25.12 | 0.05 | Genistein sulfate | M+Na | C13H24 | 39.2 | -2.11 |
| 27 | 205.19 | 28.59 | 0.05 | Daidzin 4'-O-glucuronide | M+H-H2O | C15H26O | 44 | -3.34 |
| 28 | 830.57 | 28.60 | 0.00 | Palmitic acid | M+H-H2O | C48H82NO9P | 39.4 | 0.11 |
| 29 | 473.36 | 29.96 | 0.01 | Soyasapogenol A | M-H | C30H50O4 | 38.7 | -2.49 |
| 30 | 117.02 | 32.89 | 0.00 | Methylmalonic acid | M-H | C4H6O4 | 38.4 | 4.59 |

##
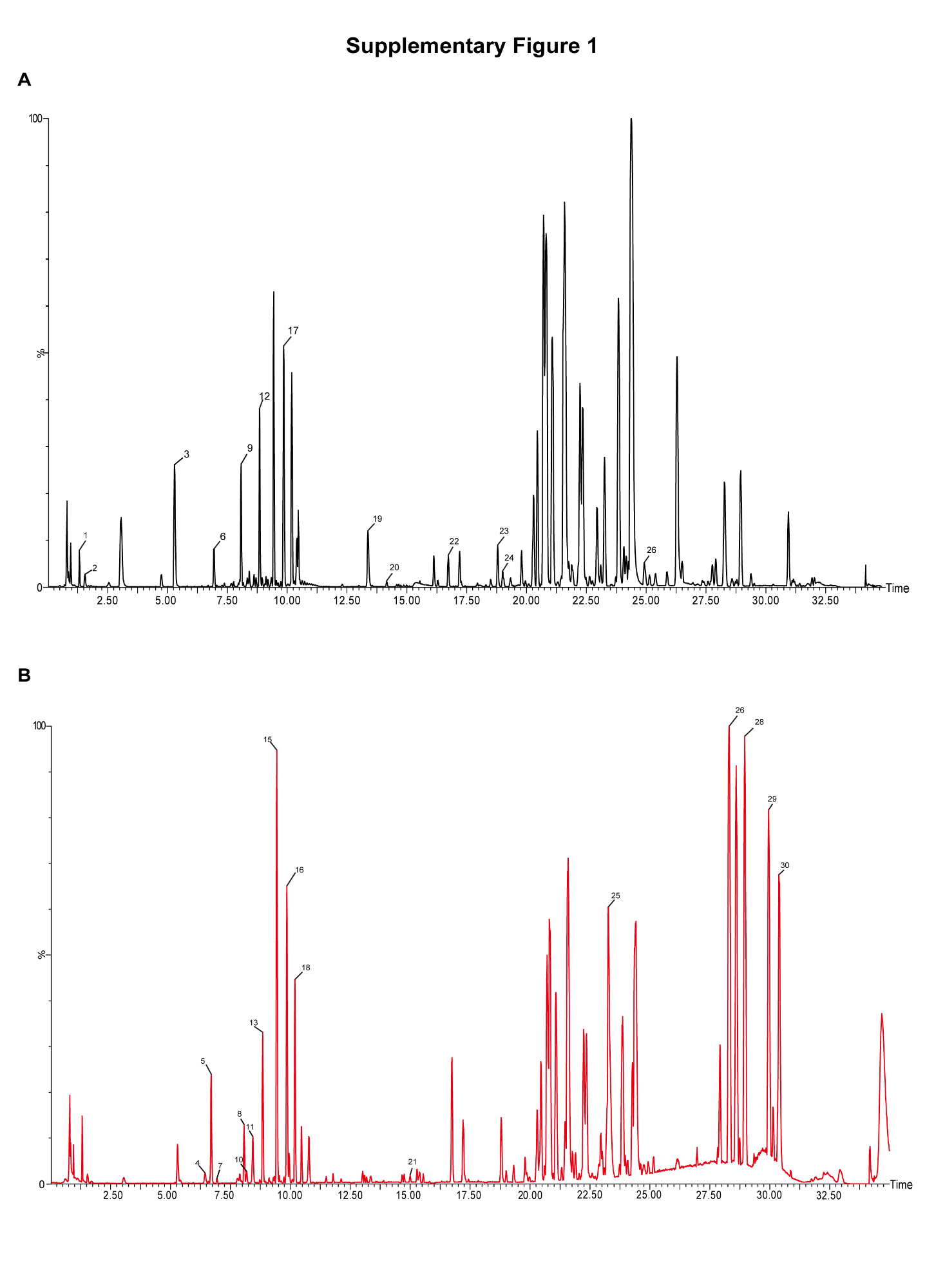
Supplementary Figures


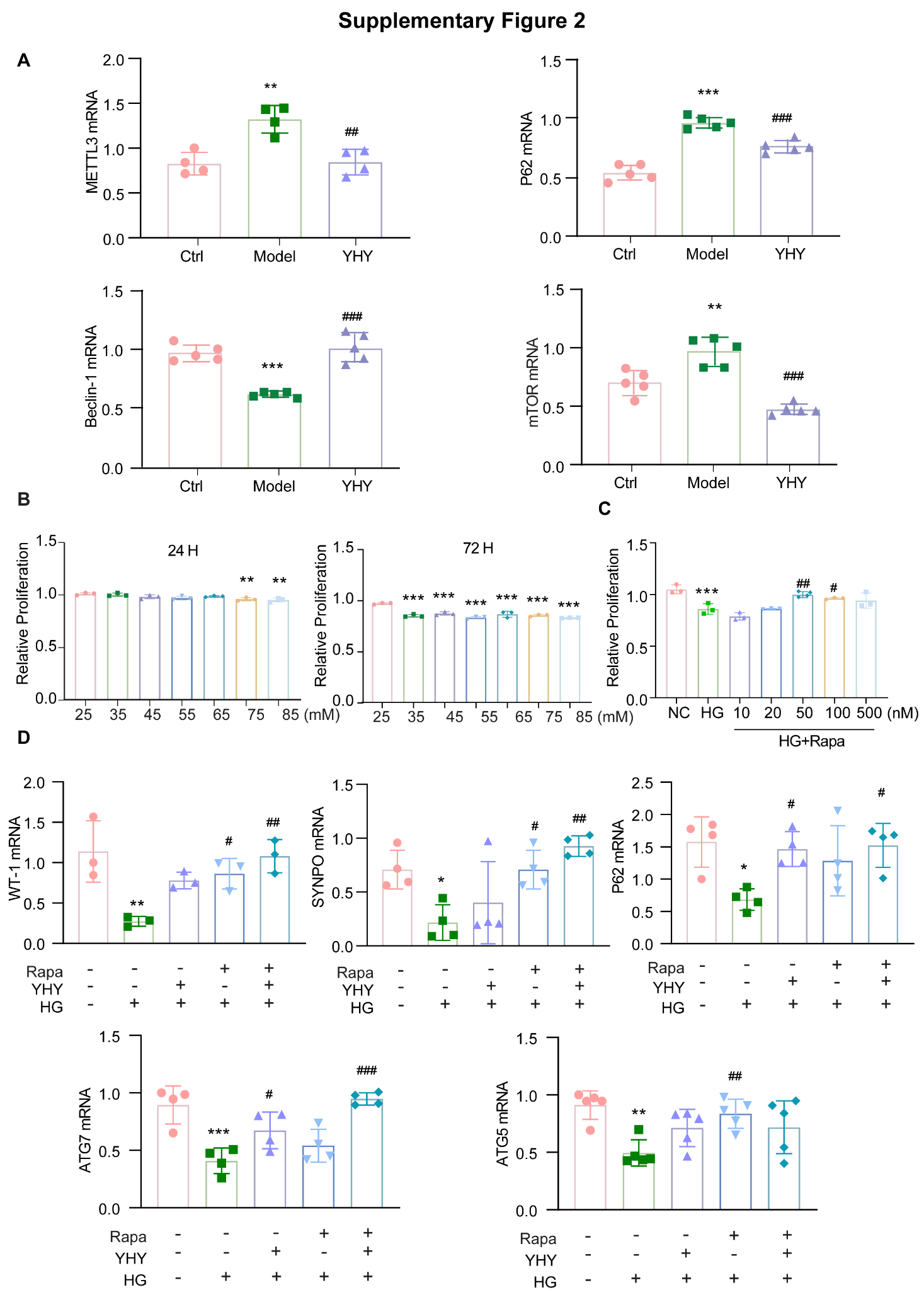

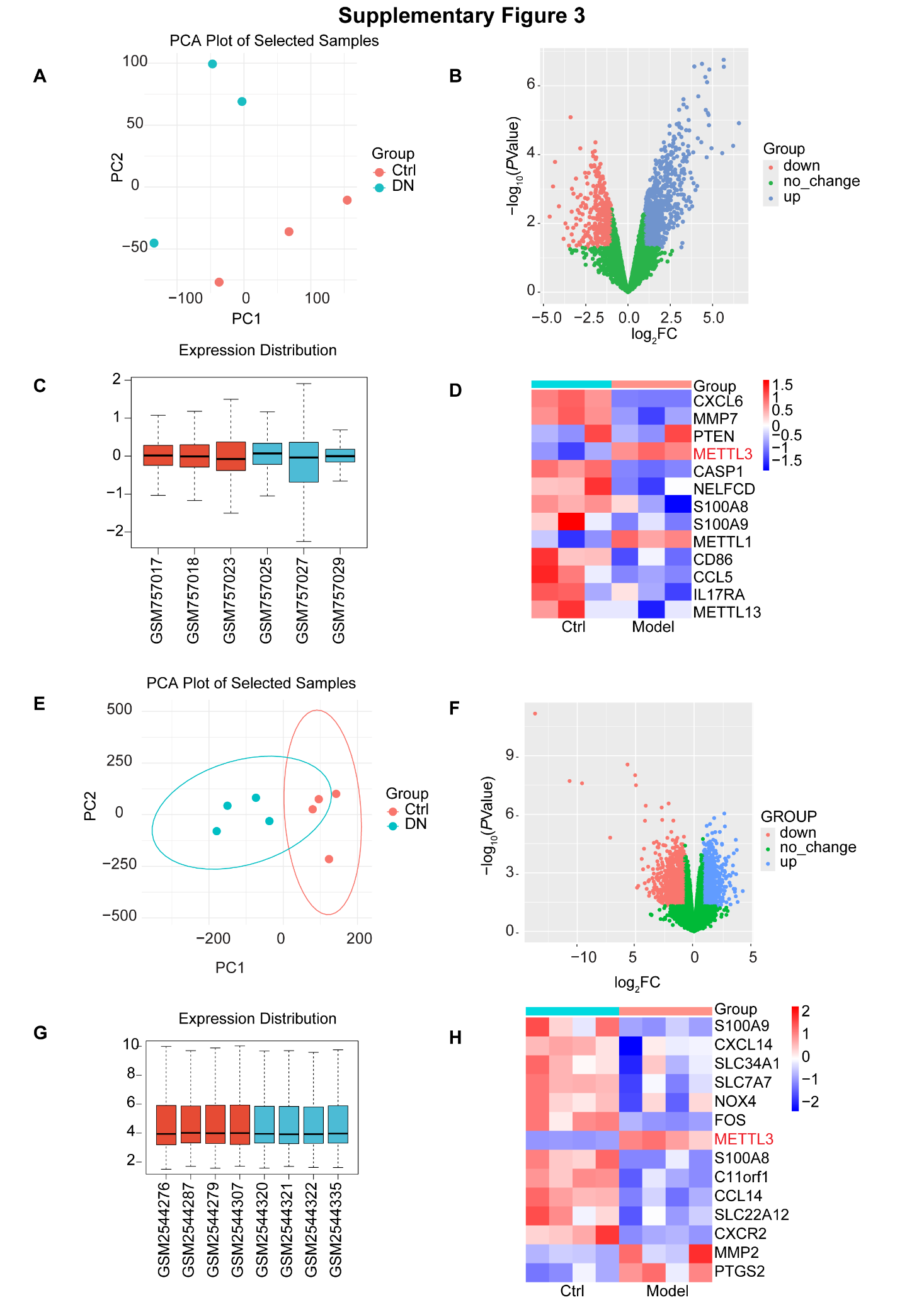

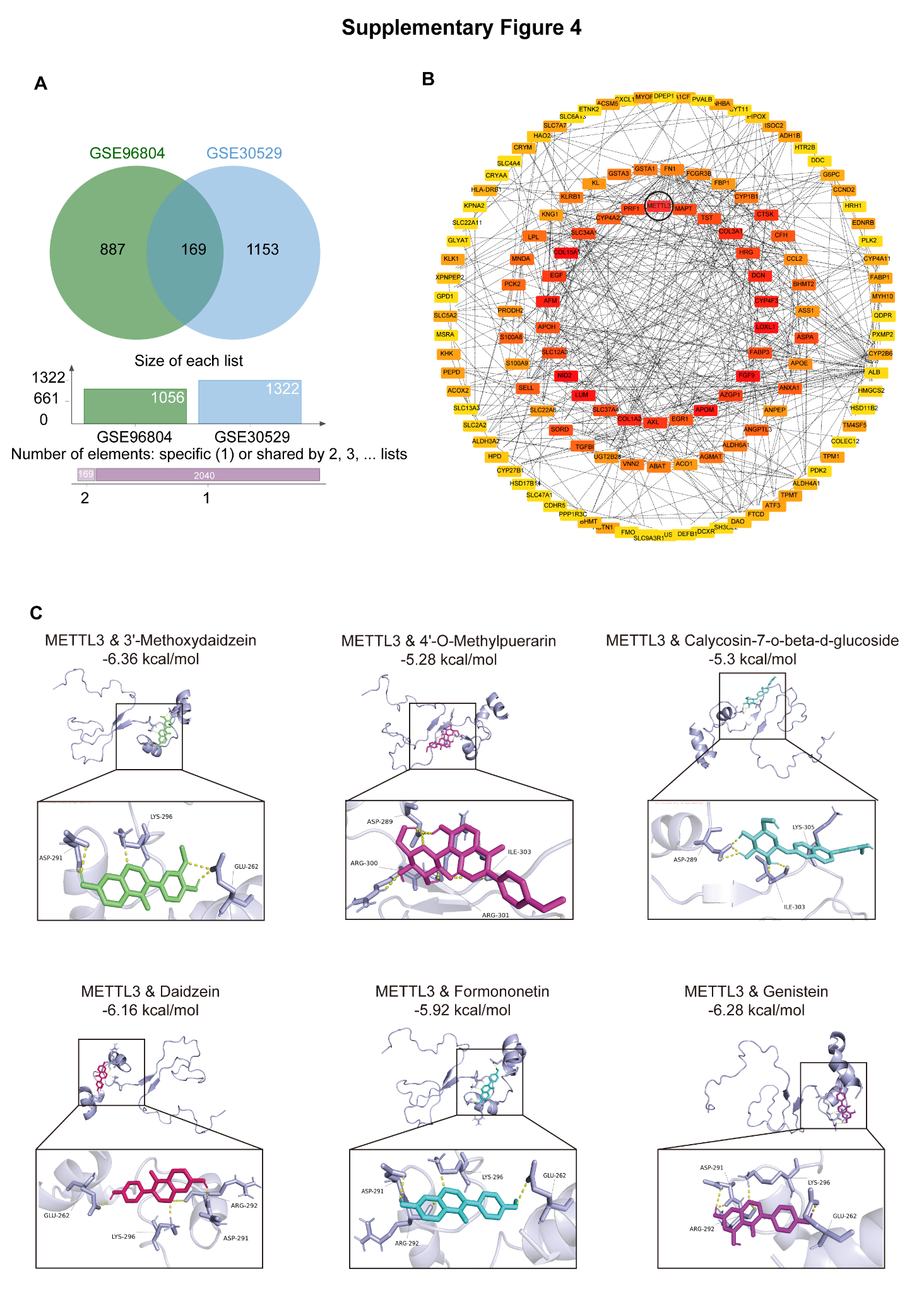

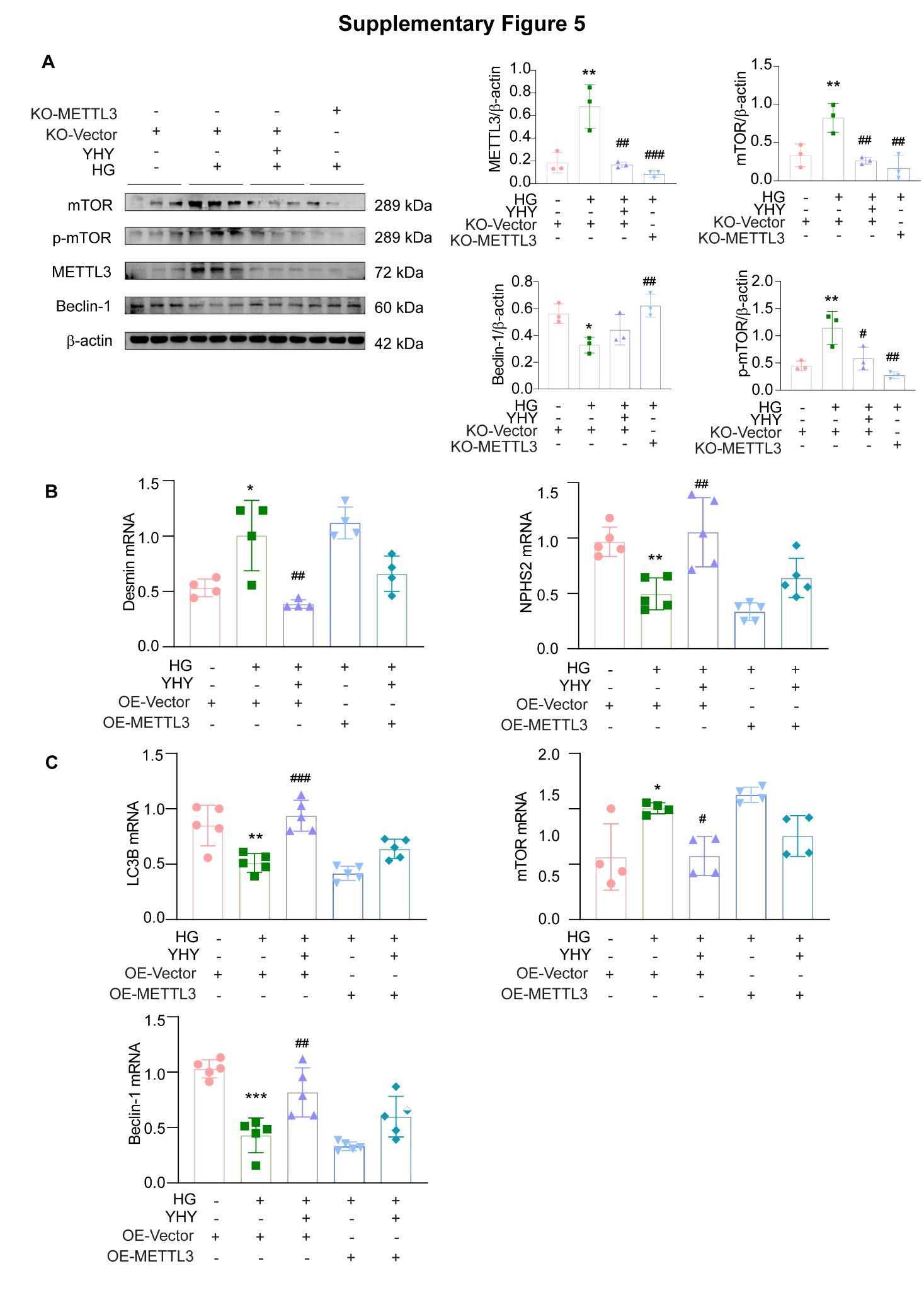


**Supplementary Figure 1.** **YHY drug-containing serum** **total ion flow diagram. (A)** The black curve denotes the total ion current for positive ions. **(B)** The red curve denotes the total ion current for negative ions.

**Supplementary Figure 2. Assessment of renal autophagic flux and rapamycin-mediated effects on podocyte injury and autophagy in *db/db* mice. (A)** mRNA expressions of autophagy-related genes (P62, Beclin-1, and mTOR) and METTL3. **(B) and (C)** CCK8: Evaluating the optimal high glucose modeling concentration and duration (24H and 72H) for MPC-5 and the administration concentration of rapamycin. **(D)** mRNA expressions of podocyte injury markers (WT-1 and SYNPO), and autophagy-related genes (P62, ATG7, and ATG5) under rapamycin intervention. Data were shown as mean ± SD, ^*^*P* < 0.05 and ^**^*P* < 0.01, ^***^*P* < 0.001 *vs.* Ctrl; ^#^*P* < 0.05, ^##^*P* < 0.01, ^###^*P* < 0.001 *vs*. Model.

**Supplementary Figure 3. Analyze the differential genes between the normal and DN sample data of clinical subjects from the GEO database. (A-H)** PCA data graph, Volcano map, Box plot and Differential gene heat map (normal samples of clinical humans *vs*. samples of DN patients in the GSE30529 database and the GSE96804 database).

**Supplementary Figure 4. Evaluation of METTL3 as a prevalent differential gene derived from GEO datasets and molecular docking with principal active chemicals of YHY. (A)** Venn Diagram (GSE96804 *vs.* GSE30529). **(B)** Network diagram of differential gene targets. **(C)** Molecular docking (METTL3 performs molecular docking with the main drug components in YHY).

**Supplementary Figure 5. The effects of METTL3 on mTOR and autophagy pathways were verified in both positive and negative ways through knockdown and overexpression in MPC-5 cells. (A)** Western blot analysis and statistical graph of mTOR, p-mTOR, Beclin-1, and METTL3 protein expression in cells knocked down with METTL3. **(B) and (C)** mRNA expressions of podocyte injury markers (Desmin and NPHS2) and autophagy-related genes (LC3B, Beclin-1 and mTOR). Data were shown as mean ± SD, ^*^*P* < 0.05 and ^**^*P* < 0.01, ^***^*P* < 0.001 *vs.* Ctrl; ^#^*P* < 0.05, ^##^*P* < 0.01, ^###^*P* < 0.001 *vs.* Model.
